# Comparative proteomic analysis of unfermented cocoa beans reveals key metabolic differences between fine-flavor and bulk genotypes

**DOI:** 10.3389/fpls.2025.1674701

**Published:** 2025-10-10

**Authors:** Ana Caroline de Oliveira, Didier Vertommen, Sébastien Pyr dit Ruys, Herve Rogez, Frédéric Debode, Dominique Mingeot, Pierre Bertin, Yordan Muhovski

**Affiliations:** ^1^ Department of Life Sciences, Unit Bioengineering, Walloon Agricultural Research Centre (CRA-W), Gembloux, Belgium; ^2^ Earth and Life Institute – Agronomy (ELI-a), Université catholique de Louvain, Louvain-la-Neuve, Belgium; ^3^ MassProt Platform, de Duve Institute, Université Catholique de Louvain (UCLouvain), Brussels, Belgium; ^4^ Integrated Pharmacometrics, Pharmacogenomics and Pharmacokinetics Group (PMGK), Louvain Drug Research Institute (LDRI), Université Catholique de Louvain (UCLouvain), Brussels, Belgium; ^5^ Centre for Valorisation of Amazonian Bioactive Compounds and Federal University of Pará, Belém, Pará, Brazil

**Keywords:** *Theobroma cacao*, unfermented cocoa beans, proteome, cocoa flavor, national cultivars

## Abstract

The organoleptic quality of cocoa beans is influenced by both post-harvest processing and genotype. Fine-flavor cocoa genotypes are especially valued for their distinctive sensory attributes, yet the proteomic differences underlying these traits remain poorly understood. In this study, we conducted a comparative proteomic analysis of unfermented beans from four *Theobroma cacao* genotypes with contrasting flavor profiles: CCN51, EET19, P7, and PA121. A total of 2,015 proteins were identified, of which 198 proteins showing significantly different abundance relative to the fine-flavor genotype EET19. Principal component analysis (PCA) effectively distinguished genotypes based on their proteomic profiles. Notably, storage proteins were more abundant in the fine-flavor genotypes, while enzymes associated with flavor precursor formation were differentially expressed. These findings provide novel insights into the proteomic basis of flavor potential in cocoa, offering targets for genotype selection and quality improvement.

## 1 Introduction

Proteomic technology has been crucial in enhancing our knowledge of the biochemical quality of industrial crops (Afzaal et al., 2022). Studies on beverages such as coffee, beer, and wine have confirmed the relevance of protein and amino acid profiles in understanding complex biological processes. These studies have emphasized the important functions of proteins and amino acids in flavor production, ripening, stress and disease resistance, and drought responses, as well as in the post-harvest management of these crops (Liu et al., 2022; Zaman and Shan, 2024). Proteomics analysis of cocoa could identify proteins that influence flavor, supporting the development of high-throughput molecular markers to assist crop improvement in breeding (Herrera-Rocha et al., 2023).

Cocoa beans are globally valued as the primary ingredient in chocolate, largely due to their distinctive flavors, which develop through a complex sequence of post-harvest steps (Santander Muñoz et al., 2019). Cocoa genotypes are generally classified as either bulk, characterized by typical cocoa aromas such as CCN51, or fine flavor, distinguished by pronounced floral and fruity notes, as found in National EET cultivars (Kadow, 2020; Qin et al., 2017; Rottiers et al., 2019).

The production of basic cocoa notes begins during the fermentation process of cocoa beans. Storage proteins and carbohydrates are degraded, producing flavor precursors such as free amino acids, hydrophilic and hydrophobic peptides, and reducing sugars (D’Souza et al., 2018; Scollo et al., 2018; Voigt and Lieberei, 2014). Storage proteins are mainly composed of albumin and vicilin (7S)-class globulin (Janek et al., 2016; Voigt et al., 1993), which provide flavor precursors, such as alanine, phenylalanine, leucine and tyrosine primarily through the activity of three endogenous enzyme groups: aspartic endoproteases, carboxypeptidases and aminopeptidases (Scalone et al., 2019; Voigt and Lieberei, 2014). After fermentation, the resulting flavor precursors are released and form the typical chocolate flavors molecules like pyrazines and furans, and some fine flavor notes like Strecker aldehydes (e.g. 2-methylbutanal, 3-methylbutanal, and benzaldeyde) by heat-induced Maillard reactions during the roasting and conching process (Collin et al., 2023; Santander Muñoz et al., 2019; Scalone et al., 2019).

In contrast to the so-called chocolate notes, additional fine flavors, such as caramel, fruity, and floral notes have been associated with the fruit pulp and the fresh cocoa cotyledons or may be developed during fermentation and drying, prior to roasting (Rottiers et al., 2019). According to previous studies, the dynamics of the production of active fine notes in cocoa are affected by a range of enzymes (Hansen et al., 2000; Scalone et al., 2019; Voigt and Lieberei, 2014) involved in the monoterpene, the L-phenylalanine pathway, the fatty acid degradation and possibly the amino acid biosynthesis (Colonges et al., 2021; 2022b).

The aroma potential of cocoa is influenced by genetic differences, which can be partially linked to variations in storage proteins and carbohydrates. Additionally, differences in the activity of endogenous enzymes present in cocoa beans can further contribute to aroma variation (Colonges et al., 2021; Hansen et al., 2000; Kumari et al., 2018; Scollo et al., 2020a). In recent years, the critical role of proteins and peptides in defining cocoa quality has intensified interest in proteomic and peptidomic studies (Herrera-Rocha et al., 2023). Scollo et al. (2018) identified over 1000 proteins in an unfermented cocoa beans cultivar. To date, only the study conducted by Scollo et al. (2020a) compared the proteins from four distinct unfermented cocoa beans genotypes (ICS1, ICS39, SCA6 and IMC67).

Previous peptidomic and proteomic studies have primarily focused on the cocoa fermentation process (Herrera-Rocha et al., 2023). However, further investigation into the type and abundance of storage and other proteins in unfermented cocoa bean genotypes, particularly those related to flavor precursor formation, can provide valuable insights into cocoa flavor quality (Rawel et al., 2019). Notably, the proteomic comparison of key reference genotypes with contrasting flavor attributes, such as the fine-flavor Nacional cultivar and the bulk-type CCN51, remains largely unexplored.

Therefore, this study aims to characterize the protein profiles of unfermented cocoa beans from genotypes with distinct organoleptic potentials, including the fine-flavor Nacional EET19, the bulk CCN51, and two Brazilian genotypes (PA121 and P7), revealing new insights into the biochemical basis of flavor development.

## 2 Materials and methods

### 2.1 Cocoa samples

Non-fermented cocoa beans from four *Theobroma cacao* genotypes (CCN51, EET19, P7 and PA121) were selected for their contrasting organoleptic attributes and used to characterize the proteomic profiles. The Brazilian genotypes P7 and PA121 were harvested at the Comissão Executiva do Plano da Lavoura Cacaueira (CEPLAC), Pará, Brazil. The Ecuadorian genotypes CCN51 (RUQ#1630) and EET19 (RUQ#1347) were kindly provided by the International Cacao Germplasm Database – ICGD, UK (http://www.icgd.rdg.ac.uk/).
These genotypes were selected to represent distinct organoleptic profiles: fine flavor (EET19), medium flavor (P7), and bulk-type flavor (PA121 and CCN51). EET19, a high-flavor Nacional genotype, was used as the reference for comparative analyses.
Three different trees of each genotype were selected for harvesting cocoa pods. Six pods were harvested from each tree. The beans were removed from the pods and stored at – 20°C.
For each biological replicate, an equal amount of beans from the six pods from a single tree was combined prior to griding to create a representative sample. This was repeated for each of the three trees per genotype, resulting in three independent biological samples for proteomic analysis. Before their grinding, the beans were freeze-dried for 24 h and subsequently ground by mortar and pestle into a fine powder using liquid nitrogen.

### 2.2 Fat and polyphenols removal

Fat and polyphenols content were removed as described in previous study (Scollo et al., 2020a). Cocoa bean powder (200 mg) was defatted three times by shaking with 4 mL of petroleum ether at 50°C for 20 min in an orbital shaker at 150 rpm followed by centrifugation at 3,700× g for 10 min. The supernatants were discarded and the precipitates were dried under vacuum. Polyphenols were extracted from the defatted powders with 3.5 mL of aqueous acetone solution (80%, v/v). The solution was vortexed for 1 min and centrifuged at 3,700× g for 10 min at 4°C. The supernatant was discarded and the extraction repeated twice. Subsequently, the powder was resuspended in 3.5 mL of ice-cold acetone, vortexed for 1 min and centrifuged as above. The samples were dried under vacuum, resulting in dried powder.

### 2.3 Protein extraction from cocoa beans

Proteins from the dried powder obtained as previously described (20 mg) were extracted with 1 ml protein extraction buffer [100mM Tris HCl, 1% dithiothreitol (DTT), 1% SDS and proteases inhibitors; pH adjusted to 8.1] (Kumari et al., 2018). The suspensions were vortexed for 1 min and subsequently extracted for 1 h at room temperature with shaking. The suspension was then centrifuged at 10.000× g for 10 min at 4°C. After extraction, sample precipitation was performed using a protocol established by our group. The supernatant containing the protein fraction was precipitated with 4 volumes of 10% TCA/acetone/0.07% DTT and kept at -20°C overnight. After centrifugation for 10 min at 14,000× g at 4°C, the precipitated proteins were washed three times with 1 mL of cold 70% acetone/0.07% DTT at 4°C, followed by 80% acetone/DTT and 90% acetone/DTT, and finally 100% acetone/0.07% DTT. Each step of wash involved a centrifugation for 10 min at 14,000× g at 4°C. The protein precipitates were solubilized in the rehydration buffer solution (7 M Urea, 2 M Thiourea, 4% CHAPS, 30 µM Tris). Proteins were quantified using the Braford protein assay (Bradford, 1976), and bovine serum albumin (BSA) as a standard. The total protein quality was determined using 12.5% sodium dodecyl sulfate-polyacrylamide gel electrophoresis (SDS-PAGE) and Coomassie staining.

### 2.4 Protein digestion

For protein digestion, Filter-Aided Sample Preparation (FASP) protocol was applied as described by Wiśniewski et al. (2009) using 30 kDa Vivacon 500 filers (Sartorius, Goettingen, Germany). Each protein sample was digested with endopeptidase rLys-C and Trypsin Gold (Mass Spectrometry grade, Promega, Madison, USA) according to the protocol described by Abid et al. (2024). The obtained peptides from each sample were resuspended in 3% (v/v) acetonitrile with 0.1% (v/v) trifluoroacetic acid (TFA) before the mass spectrometry analysis.

### 2.5 Protein LC-MS/MS analysis

The peptides were analyzed by nanoLC-MS/MS on an Orbitrap Fusion Lumos tribrid mass spectrometer (ThermoFisher Scientific, Waltham, USA) at the MASSPROT platform of the De Duve Institute (Brussels, Belgium). The peptides were subjected to NSI source followed by tandem mass spectrometry (MS/MS) in Fusion Lumos, which was coupled online to the nano-LC. Peptides were directly injected onto a reversed-phase pre-column (Acclaim PepMap 100, Thermo Scientific, Waltham USA) and eluted using backflush mode. Intact peptides were detected in the Orbitrap at a resolution of 120,000. Peptide separation was performed using a reversed-phase analytical column (Biozen Peptide Polar C18 SL, 250 x 0.075 mm, Phenomenex) with a linear gradient of 4%-32% solvent B (0.1% FA in 80% ACN) for 100 min, 32%-60% solvent B for 10 min, 60%-95% solvent B for 1 min and holding at 95% for the last 10 min at a constant flow rate of 300 nL/min on an Ultimate 3000 RSLC system.

Peptides were selected for MS/MS using HCD setting at 30; ion fragments were detected in the linear IonTrap. Ions exceeding a threshold intensity of 5.0 × 10³ in the MS survey scan were selected for data-dependent procedure. The instrument operated in a top-speed mode, alternating between one MS scan and MS/MS scans for a cycle time of 3 seconds. Dynamic exclusion was enabled with a duration of 60 seconds. The electrospray voltage applied was 2.1 kV. MS1 spectra were obtained with an AGC target of 4E5 ions and a maximum injection time of 50ms, MS2 spectra were acquired with an AGC target of 1E4 ions and a maximum injection time set to 35ms. For MS scans, the *m/z* scan range was 375 to 1800. The mass spectrometry proteomics data were deposited to the ProteomeXchange Consortium (http://proteomecentral.proteomexchange.org) via the PRIDE partner repository (Perez-Riverol et al., 2022) with the dataset identifier PXD045216 and 10.6019/PXD045216.

### 2.6 Protein identification and quantification

All MS/MS data were processed using Sequest HT search engine within Proteome Discoverer 2.5 SP1 software against the protein sequence database of *Theobroma cacao* obtained from UniProt database (https://www.uniprot.org/, accessed 02 February 2024), with 40.608 entries. The trypsin (RK) was specified as cleavage enzyme allowing up to 2 missed cleavages, 4 modifications per peptide and up to 5 charges. Mass error was set to 10 ppm for precursor ions and 0.3 Da for fragment ions. Oxidation on Met (+15.995 Da), Carbamidomethyl on Cys (+57.021 Da), pyro-Glu formation from Gln or Glu (-17.027 Da or – 18.011 Da respectively), Acetylation (+42.011Da) and Met-loss (-131.040 Da) on protein-terminus were considered as variable modifications. False discovery rate (FDR) was assessed using Percolator and thresholds for protein, peptide and modification site were specified at 1%. For proteomic analysis, only proteins with a minimum of two unique peptides and peptide spectrum matches (PSMs) > 3 were used. The Protein Discoverer software was used to obtain label-free quantification by the area under the curve (AUC) for each peptide and aggregate it into proteins. Normalization was done on the total protein content. The abundance ratio (fold-change) between the EET19 versus the others genotypes of any protein was obtained using the Protein Discoverer software by comparing the average abundance values of the three biological replicate for each genotype. Abundance ratios above or below a predefined threshold and with an adjusted p-value < 0.001 were used to define the differentially abundant proteins (DAPs). The abundance ratio threshold was set between (0.5 and 2.0), the proteins expressed with values less than 0.5 were considered down-regulated and the proteins expressed with values greater than 2.0 were up-regulated.

### 2.7 Bioinformatics and data analysis

The function of identified proteins was assessed using the Universal Protein Resource (UniProt). Functional annotations of DAPs were carried out in the PANTHER server (http://www.pantherdb.org/) using GO annotations of *T. cacao* as a reference, accessed 10 December 2024. The proteins were classified based on four categories: molecular function, biological process, cellular component and protein class. Hierarchical clustering analysis was performed with euclidean distance and complete linkage using *complexheatmap* package from RStudio (R Core Team, 2024). In addition, a principal component analysis (PCA) was obtained using Proteome Discoverer software to identify the abundance protein differences between the cocoa beans.

## 3 Results

### 3.1 Overview of the proteomic profiles from all cocoa genotypes

To compare the cocoa proteomic analyses were performed on unfermented beans from four different *Theobroma cacao* genotypes. Three biological replicates were prepared and subsequently analyzed by nanoLC-MS/MS. A total of 2015 proteins were identified in the four genotypes with a FDR < 1%. Among them, 1724 were filtered to a minimum 1 unique peptide and 4 PSMs. The complete list of identified proteins in all genotypes is provided in **Supplementary Table S1**.

Several identified proteins were genotype-specific: 3, 3, 4 and 1 proteins were only detected in the genotypes EET19, CCN51, P7 and PA121, respectively. Conversely, a total of 64, 32, 30 and 49 proteins were not detected in the genotypes EET19, CCN51, P7 and PA121, respectively (**Supplementary Table S2**).

The top-ranked proteins, based on summed posterior error probability (PEP) scores, are listed in **Supplementary Table S3**. The five most abundant proteins were vicilin-A (A0A061EM85), lipoxygenase (A0A061F2P1), pyruvate phosphate dikinase (A0A061EMS0), 21 kDa seed protein (A0A061G2K6) and pyrophosphate-fructose 6-phosphate 1-phosphotransferase subunit alpha (A0A061FEW0). These proteins were consistently abundant in all four genotypes.

### 3.2 Identification of differentially abundant proteins from the four cocoa genotypes

**Figure 1:**
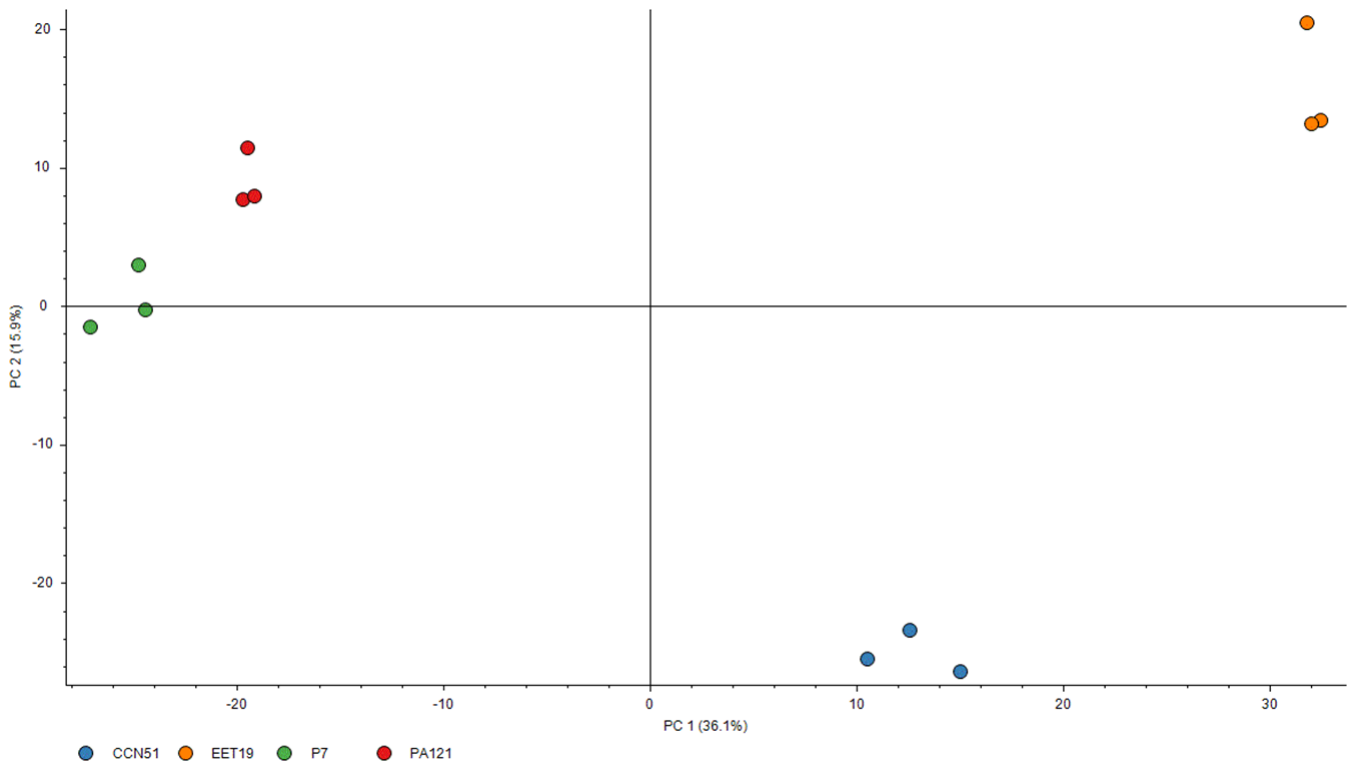
PCA score plot of all identified proteins with FDR < 1% and minimum 1 peptide and 4 PSMs. Biological replicates of the same genotype are displayed with the same color.

To assess whether the proteomic data enabled differentiation between genotypes based on protein abundance, abundance ratio weight vs abundance ratio (log) plot was generated to identify differentially abundant proteins between EET19 and each of the other’s genotypes. In the comparative proteomics analysis, a stringent threshold of at least 2-fold increase (≥ 2.0) or a 2-fold decrease (≤ 0.5), alongside an adjusted *p*-value ≤ 0.001 was applied to define biologically significant up- and down-regulated proteins. A graphical summary of these comparisons is provided in **Supplementary Figure S1**. PCA was then conducted using the DAPs as variables and the genotypes as observations. The PCA score plot of the first two principal components revealed clear separation among the four cocoa genotypes, with PC1 and PC2 accounting for 36.1% and 15.9% of the total variance, respectively (**Figure 1**).

**Figure 2:**
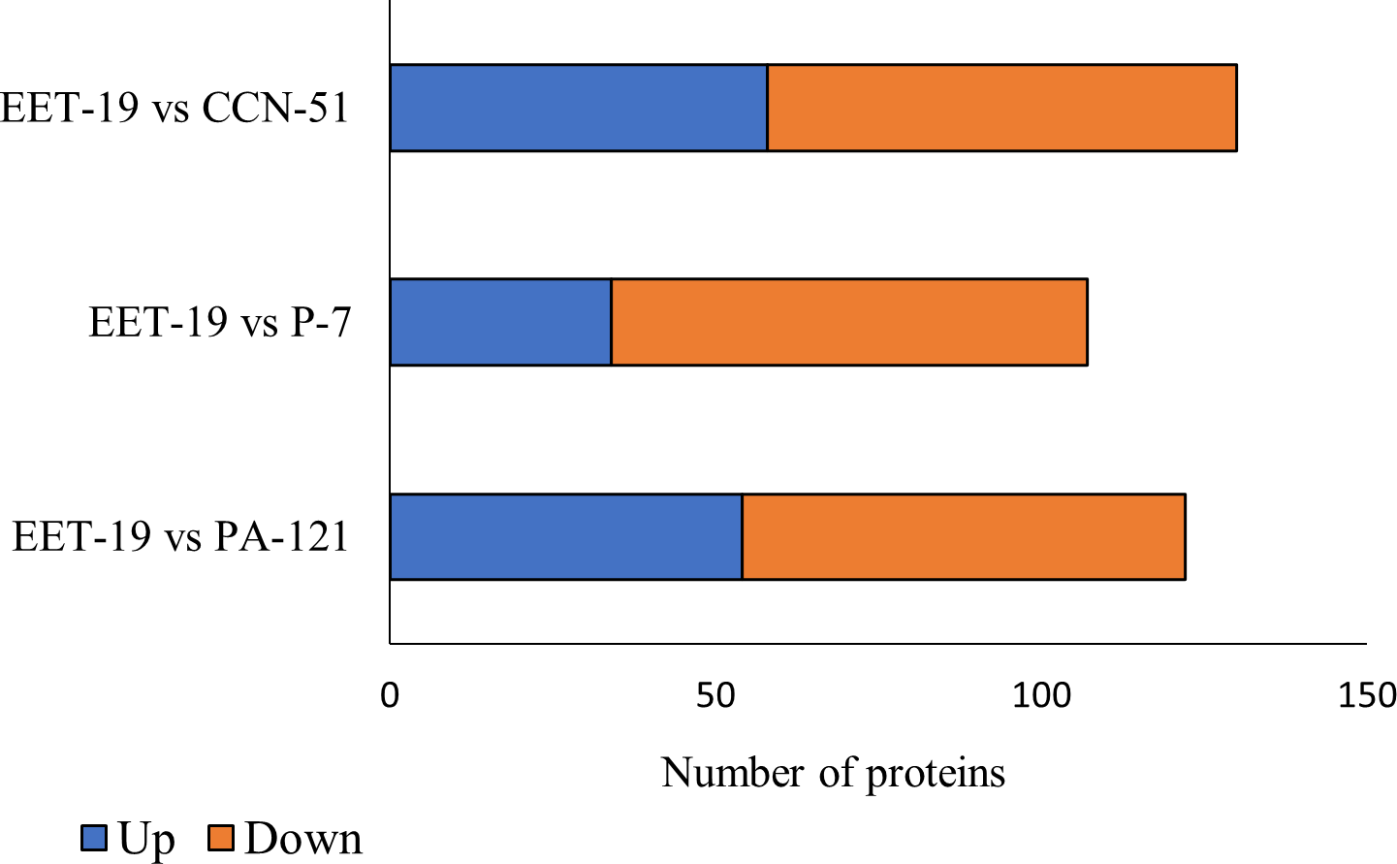
Differentially abundant protein (DAPs) with abundance ratio adj. *p*-value ≤ 0.001 from pairwise comparison between the EET19 and each of the other genotypes.

Pairwise comparisons between EET19 and the other genotypes revealed substantial variability in protein abundance, with 198 proteins showing statistically significant differences (adj. *p*-value ≤ 0.001) in at least one comparison. A complete list of differentially abundant proteins (DAPs), including their direction of regulation and fold change for each comparison, is presented in **Supplementary Table S5**. A total of 130 proteins were determined to be differentially expressed in the genotype EET19 compared to CCN51, of which the abundance of 58 proteins increased while the abundance of the other 72 decreased. In the genotype EET19 compared to P7, 107 proteins were determined to be differentially expressed, of which the abundance of 34 proteins increased while the other 73 decreased. In the genotype EET19 compared to PA121, 122 proteins were determined to be DAPs, of which 54 increased while the other 68 decreased (**Figure 2**).

### 3.3 Functional annotation of DAPs

**Figure 3:**
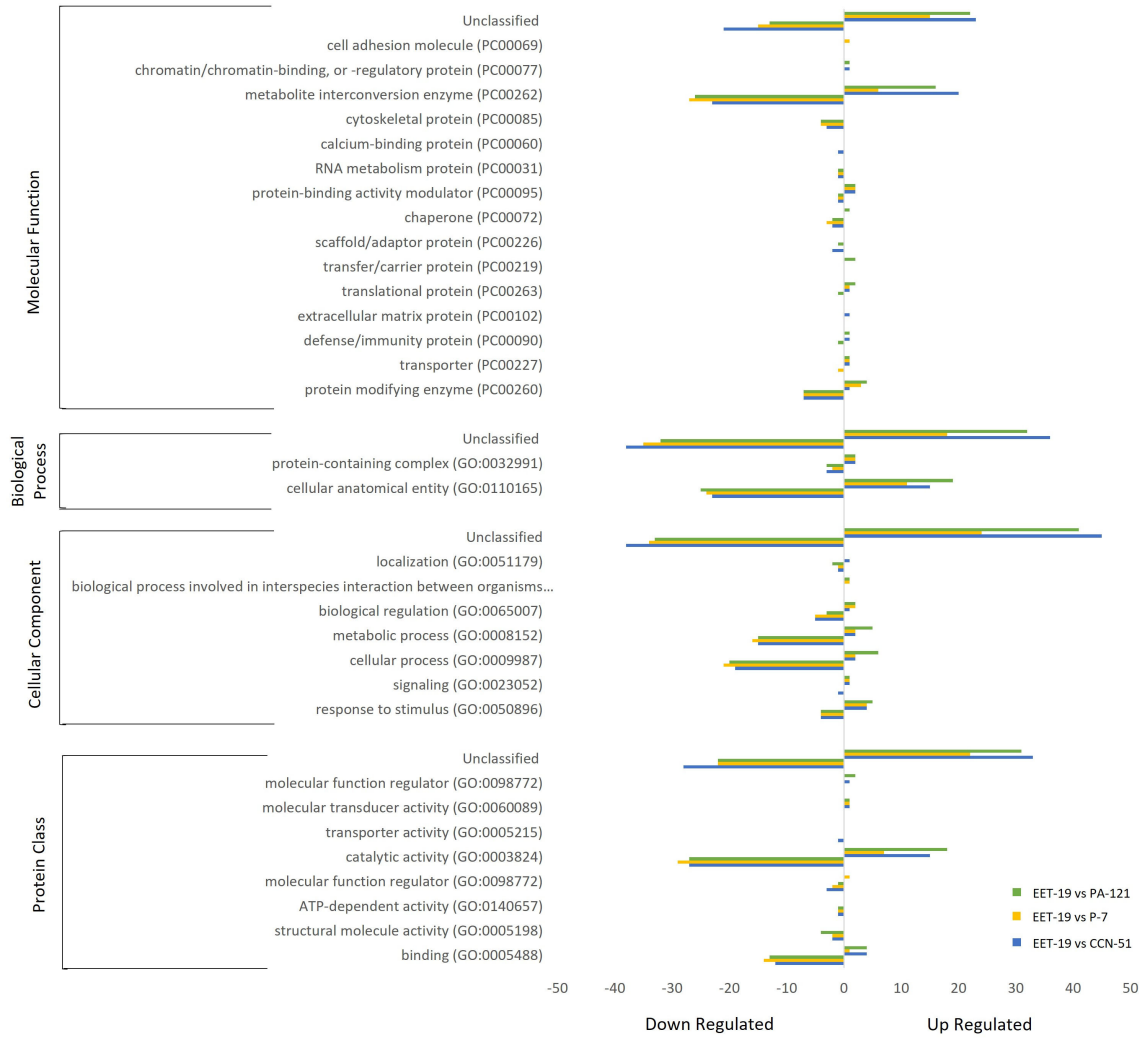
Classification of differentially expressed proteins identified by LC-MS/MS across four cocoa genotypes. Up- and down-regulated proteins were categorized into molecular function, biological process, cellular component and protein class categories. Down-regulated proteins are plotted to the left, and up-regulated protein to the right, of the central *Y*-axis. The values indicate the number of proteins in each functional category.

A graphical representation of the 198 up- and down-regulated proteins classifications according to the Panther database (http://www.pantherdb.org/) is shown in **Figure 3**. These proteins were categorized into 16 molecular function groups, 3 biological processes, 8 cellular components and 9 protein classes. The results indicate that the differentially abundant proteins in all genotypes were involved in diverse biological processes and molecular functions. Proteins with unclassified functions represented the largest group in all categories. The majority of those proteins were found in the orthologs with catalytic activity. Following the unclassified proteins, most DAPs were involved in molecular function such as catalytic activity (33.8%) and binding (15.9%). These categories contained a higher number of down-regulated proteins compared to up-regulated ones. In EET19, most proteins linked to biological process were classified as cellular and molecular processes, representing 17.8% and 13.9% of the total number of DAPs up- and down-regulated, respectively. Within protein classes, metabolite interconversion enzyme accounted for the largest category, comprising 38.2% of DAPs.

### 3.4 Genotype-related proteins according to different cocoa metabolic pathways

**Figure 4:**
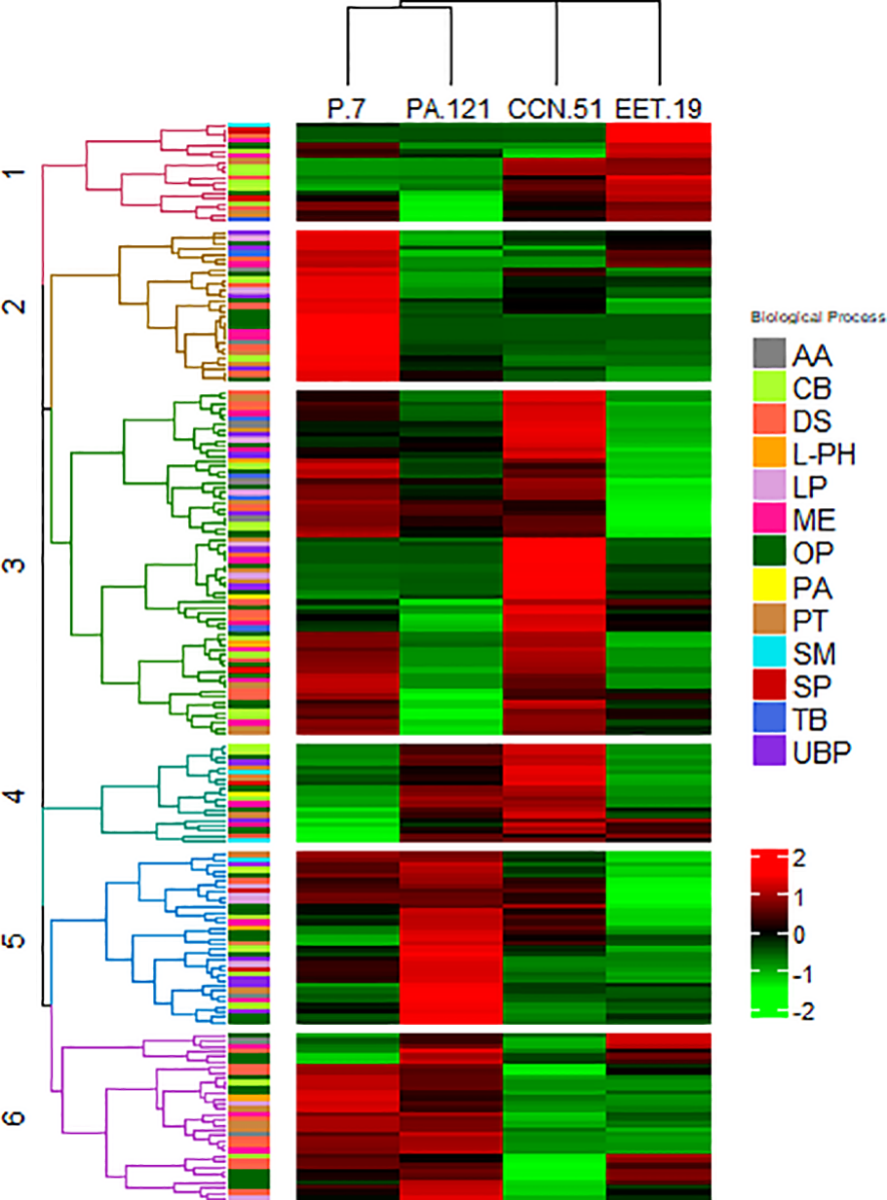
Hierarchical Cluster Analysis (HCA) representing the proteins with significant different abundances of the cocoa genotypes from the proteome analysis. The heatmap was divided into six principal clusters. Proteins from clusters 1 to 6 obtained by HCA are presented in **Supplementary Table S6** and in the dendrograms shown in **Supplementary Figure S2**. Each protein in the HCA is color coded based on their major biological process: Amino acid biosynthesis (AA), Carbohydrate metabolic process (CB), Defense and stress (DS), L-phenylalanine degradation (L-PH), Lipid metabolic process (LP), Metabolism and energy (ME), Others metabolic process (OP), Proanthocyanin biosynthesis (PA), Protein metabolic process (PT), Secondary metabolism (SM), Storage protein (SP), Terpenoid biosynthesis (TB), Unspecified biological process (UBP).

Hierarchical clustering of the 198 DAPs based on their Z-scored protein abundances revealed distinct differences among the cocoa genotypes. The DAPs were grouped into six principal clusters (**Figure 4**). The cluster 1 (**Figure 4**, in red) contained a total of twenty-seven proteins including storage proteins and proteins involved in flavor precursors formation of which 19 (9.6%) were up-regulated in EET19. The clusters 2 to 6 (**Figure 4**) contained 29, 66, 19, 33 and 32 proteins representing 14.6, 33.3, 9.6, 16.7 and 16.2% of the total DAPs.

The 198 identified DAPs were classified into 13 functional categories related to key cocoa metabolic pathways involved in flavor release, defense and stress responses, and metabolism and energy based on literature and Uniprot *in silico* analysis (https://www.uniprot.org/, accessed on 15 June 2024) (**Supplementary Table S4**; **Figure 4**). These categories include: storage proteins (6), protein metabolic processes (22), amino acid biosynthesis (29), carbohydrate metabolic processes (25), proanthocyanin biosynthesis (3), L-phenylalanine degradation (6), terpenoid biosynthesis (5), secondary metabolic biosynthesis (3), lipid biosynthetic and metabolic processes (13), defense and stress (29), metabolism and energy (19), others metabolic process (44), unspecified biological process (16). A summary of the main identified DAPs is provided in **Supplementary Table S5**.

Among the 198 identified DAPs, five were classified as storage proteins. Notably, three 21 kDa seed proteins (A0A061FWL5, A0A061E5P2 and A0A061G2L0) were up-regulated in EET19, with A0A061E5P2 detected exclusively in this genotype.

Our study identified thirteen proteins involved in protein degradation and amino acid biosynthesis that were up-regulated in EET19 compared to at least one other cocoa genotype. Among these, three proteins with carboxypeptidase activity (A0A061EFD5, A0A061GQ08, A0A061FIY5), were up-regulated in EET19 compared to the other genotypes. In contrast, the carboxypeptidase A0A061H0B2 was consistently down-regulated in EET19 compared to all genotypes.

A total of twenty-five proteins were classified as involved in carbohydrate metabolism, with eight, six and nine proteins up-regulated in EET19 compared to CCN51, P7 and PA121 genotypes, respectively. Whereas four proteins were found at a significant higher level in the cocoa genotype P7 compared to others.

Four proteins involved in secondary metabolite biosynthesis were up-regulated in EET19 genotype. Interestingly, proteins associated with terpene biosynthesis and L-phenylalanine degradation were down-regulated in the National cocoa EET19 but found in higher level in the high flavor cocoa genotype P7 compared to other genotypes. These proteins include the (+)-delta-cadinene synthase (A0A061GGG2), 3S-linalool/(E)-nerolidol/(E,E)-geranyl linalool synthase (A0A061GF49), 4-hydroxy-3-methylbut-2-enyl diphosphate synthase isoform 1 (A0A061GYL3), terpene cyclase/mutase family member (A0A061FR70), cytochrome P450 (A0A061ENM7), cinnamoyl CoA reductase 1 isoform 1 (A0A061G9C7) and short chain alcohol dehydrogenase (A0A061E210). In contrast, the leucoanthocyanidin dioxygenase isoform 1 (A0A061G577) and benzoyl coenzyme A: benzyl alcohol benzoyl transferase (A0A061F0U3) were more abundant in the PA121 genotype.

Among enzymes involved in the L-phenylalanine pathway, cinnamyl alcohol dehydrogenase (CAD), cinnamoyl CoA reductase 1 isoform 1, short chain alcohol dehydrogenase (ADH) were identified. CAD is the first enzyme in this pathway capable of removing hydrogen from cinnamyl alcohol to convert it into cinnamaldehyde or degrade benzaldehyde to benzyl alcohol (Colonges et al., 2021).

In our study, four proteins involved in the lipid biosynthetic and metabolic process were up-regulated in EET19 compared to at least one other genotype. In contrast, two proteins were found at a significantly higher level in the CCN51 compared to the other genotypes. Additionally, lipoxygenase (A0A061F3Q9) and cytochrome B5 (A0A061EKX2) were detected at significantly higher levels in the P7 genotype compared to the others.

The highest abundance of proteins associated with defense and stress mechanisms was observed in the EET19 genotype. Interestingly, some proteins such as peroxidase are important in reducing astringency during fermentation process and enhancing the organoleptic quality of cocoa beans. However, several proteins were absent in EET19, including the HSP20-like chaperones superfamily protein (A0A061GXF3 and A0A061GXF6), glutathione peroxidase (A0A061G576), heat shock protein 90.1 isoform 1 (A0A061G8E4), phosphoenolpyruvate carboxylase family protein isoform 5 (A0A061EJV5). In contrast, the protein plasmodesmata callose-binding protein 5 (A0A061DNF1) was exclusively detected in the CCN51 genotype.

Most of the proteins classified under metabolic and energy-related functions were found at higher levels in the P7 genotype.

## 4 Discussion

A total of 2015 proteins were identified in the unfermented cocoa beans from four genotypes with contrasting flavor profiles. In comparison, Scollo et al. (2020a), identified 430 proteins, of which 61 were found to be significantly differentially expresses among four cocoa genotypes. Among the genotypes used here, CCN51 and Forastero PA121 are classified as bulk flavor types. Despite its recognized inferior organoleptic quality, CCN51 is recognized as a precocious cultivar with strikingly high cocoa butter content (54%), making it a highly productive genotype for the cacao butter industry (Boza et al., 2014; De Wever et al., 2020). Additionally, this genotype is significantly more productive and disease-resistant than National cultivars. Although the CCN51 is traditionally classified as a bulk-flavor genotype, sensorial analysis performed for CCN51 liquor revealed the presence of slight notes of fine flavor like floral and fruit aroma, but not enough to be considered a fine flavor genotype (Boza et al., 2014). The National EET19, cultivated exclusively in Ecuador, is considered a fine-flavor cocoa highly valued by chocolate manufacturers and consumers for its intense floral aroma, commonly referred to as Arriba (Rottiers et al., 2019). The Brazilian genotype P7 is classified as having a medium-flavor. Both Brazilian genotypes (PA121 and P7) were selected for their economic relevance and widespread cultivation in the Amazon region.

The top-ranked proteins identified in the present study offer a representative overview of the proteomic profiles across all genotypes. These include vicilin-A, 21 kDa seed protein albumin, lipoxygenase, pyruvate phosphate dikinase, and pyrophosphate-fructose 6-phosphate 1-phosphotransferase subunit alpha. Herrera-Rocha et al. (2023) reviewed research on cocoa bean proteins and highlighted the vicilin, albumin, lipoxygenase, RmlC-like, and glyceraldehyde-3-phosphate dehydrogenase C2 (GAPDH) as the top five peptide source proteins in cocoa.
The top-scored proteins, 21 kDa seed albumin and vicilin A, are recognized as storage proteins. Among the last top five scored proteins, lipoxygenase is involved in the fatty acid pathway and responsible for many of the volatiles including esters, aldehydes and alcohols such as 2-hexanol (Colonges et al., 2022a; Rottiers et al., 2019). Pyruvate phosphate dikinase is a vital enzyme in cellular energy metabolism catalyzing the ATP- and P_i_ -dependent formation of phosphoenolpyruvate from pyruvate in C_4_ -plants (Mingesxet al., 2017). The process of starch biosynthesis and seed development in rice is regulated by the enzyme pyrophosphate-fructose 6-phosphate 1-phosphotransferase (Chen et al., 2020).

The four cocoa beans genotypes studied here exhibited distinct protein abundances, encompassing storage proteins, enzymes and structural proteins. The high-flavor National cocoa genotype EET19 showed elevated levels of storage proteins, whereas CCN51 displayed a higher abundance of specific enzymes, notably those involved in lipid biosynthesis. Kumari et al. (2018) reported a remarkably high protein content in CCN51 in comparison with other cocoa genotypes including National EET and COMUM types from Ecuador and Brazil.

Deus et al. (2020) analyzed the amino acid profiles in chocolates made from different commercial cocoa genotypes and processed under identical conditions. Their findings highlighted the prevalence of hydrophobic over acidic amino acids for genotype CCN51 compared to the fine flavor Brazilian genotype Catongo, with ratio of ~3.6 (hydrophobic/acidic). Interestingly, compounds associated with desirable chocolate notes have been found in higher concentrations in CCN51 than in the Nacional cultivars (Kadow et al., 2013; Rottiers et al., 2019). These observations align with the higher abundance of enzymes in CCN51 identified in this study, which are responsible for generating flavor precursors and facilitating the Maillard reaction during processing.

However, cocoa beans from CCN51 are known to have a high content of undesirable off-flavors and accompanied by strong acidity, bitterness and astringency due to the presence of nonvolatile compounds such as phenolic compounds, in particular proanthocyanidins and flavonoids (Rottiers et al., 2019; Santander Muñoz et al., 2019). The high concentration of these compounds can mask the subtle volatile fine flavors notes detected in CCN51 (Colonges et al., 2022c). In contrast, the National EET cultivars are characterized by higher concentration of fruity and floral volatile compounds and lower levels of undesirable off-flavors, bitterness and astringency, although variation exist among EET cultivars (Rottiers et al., 2019).

Phenolic compounds degrade during fermentation, which reduces the bitterness and astringency of cocoa beans. Anthocyanins are hydrolyzed to anthocyanidins and sugars (galactose and arabinose) by glycosidases, while polyphenol oxidases convert the polyphenols (mainly epicatechin and free anthocyanidins) into quinones (Santander Muñoz et al., 2019). In the present study, the peroxidases were detected at higher levels in the EET19 genotype. These enzymes are associated with polyphenol oxidation and have been implicated in the reduction of astringency and bitterness of this genotype (Herrera-Rocha et al., 2023). This enzymatic activity may partially explain why, despite the slight presence of fine-flavor volatile notes in CCN51, it remains categorized as a bulk genotype in sensory evaluations.

The biosynthesis of volatile compounds in cocoa fruity traits is a very complex process involving multiple pathways. Flavor precursors are produced during fermentation by proteolysis from storage proteins and carbohydrates degradation and, posteriorly chocolate flavor notes are formed by Maillard reactions during roasting and conching, leading to the development of characteristic chocolate flavor notes. Therefore, cocoa aroma arises from two major biosynthetic sources: i) chocolate flavor and aldehydes-Strecker compounds from the non-enzymatic reaction, namely Maillard reactions, between the flavor precursors (reducing sugars and free amino groups), and ii) fine flavor notes from the monoterpene pathway, the L-phenylalanine pathway, the fatty acid degradation pathway and possibly amino acid biosynthesis pathway (Colonges et al., 2021; 2022b).

During Maillard reactions, the degradation of each specific amino acid produces a unique Strecker aldehyde with desirable cocoa/chocolate notes (Voigt and Lieberei, 2014). However, important fine flavor compounds in typical roasted cocoa such as acids, esters and alcohols are not formed from flavor precursors during roasting but delivered by fermented unroasted cocoa (Santander Muñoz et al., 2019). The largest category of fruit and floral aroma in cocoa are benzenoid compounds such as benzaldehyde, benzyl alcohol, 1-phenylethyl acetate, ethyl benzoate, benzyl benzoate, and acetophenone, which are mainly synthesized via the L-phenylalanine biosynthetic pathway (Colonges et al., 2021; 2022a). In many flowers and fruits, valine, leucine, and isoleucine precursors can also produce aroma, such as branched-chain aldehydes, alcohols, and esters through the amino acid biosynthesis and degradation (El Hadi et al., 2013). Another category of the volatile straight-chain aldehydes, alcohols, ketones, acetate esters and fatty acids esters in aroma components are synthesized by the fatty acid pathway (El Hadi et al., 2013). Floral aromas such as linalool, epoxylinalool, and acetophenone are produced via the monoterpene biosynthetic pathway (Colonges et al., 2021).

The DAPs identified in this study were classified according to the principal cocoa metabolic pathways associated with the flavor release, defense and stress response, and energy metabolism, as established in published literature. The functional roles of these DAPs were analyzed and discussed below within the context of key metabolic pathways involved in flavor development. These include: i) storage proteins, ii) protein metabolism and amino acid biosynthesis, iii) carbohydrate metabolism, iv) proanthocyanin biosynthesis, v) L-phenylalanine degradation, vi) terpenoid biosynthesis, vii) secondary metabolite biosynthesis and viii) lipid biosynthetic and metabolism.

Three 21 kDa seed proteins were identified at a significantly higher level in EET19 compared to the other genotypes. These proteins are recognized as key storage proteins involved in the formation of flavor precursors. Most of the peptides are formed predominantly from the proteolysis of the vicilin and 21 kDa albumin (Scollo et al., 2020a). Scollo et al. (2020b) identified two 21 kDa seed albumins, a storage protein, present at a significantly higher level in the genotype ICS39 compared to IMC67 further confirming their role as abundant storage proteins with potential implications in cocoa flavor development.

Protein degradation in cocoa beans is primarily mediated by endoproteases, aminopeptidases, and carboxypeptidases, which show significant differences in activity across unfermented cocoa beans from different genotypes with contrasting flavor profiles (Voigt and Lieberei, 2014). For example, Hansen et al. (2000) reported that an unfermented high fine flavor genotype PA7 contained the highest carboxypeptidase, endoprotease and aminopeptidase activities. In contrast, the authors observed that beans of the low cocoa flavor genotype UIT1 contained low carboxypeptidase activity, showing that this particular genotype exhibited low activity for all three proteases (Hansen et al., 2000).

In a previous study, the serine carboxypeptidase was identified at a significantly higher level in ICS39 genotype, which has a caramel and nutty flavor (Scollo et al., 2020a). Similarly, Der Agopian et al. (2020) found in ethylene-treated papayas that the enhancement of volatile compound production was associated with increased amino acids level due to upregulation of proteases and peptidases, including putative serine carboxypeptidase-like 53 and subtilisin-like serine endopeptidase.

Carboxypeptidase is an exopeptidase that cleaves C-terminal amino acids from mainly hydrophobic oligopeptides formed by the action of aspartyl protease during fermentation with the preferential release of hydrophobic amino acids and peptides from globulin and acidic amino acids from albumin (Deus et al., 2020). This enzymatic action preferentially releases hydrophobic amino acids (e.g., phenylalanine, alanine and leucine) from globulin storage proteins, whereas acidic amino acids (e.g. glutamic acid and aspartic acid) are released very slowly from albumin fraction, suggesting that most peptides liberated in cocoa beans should not have acidic C-terminal amino acids. Although hydrophobic amino acids dominate cleavage products, mass spectrometry analyses confirm that peptides derived from cocoa vicilin (7S globulin) and 21 kDa albumin can contain acidic C-terminal residues, such as glutamic acid and aspartic acid (Scalone et al., 2019; Voigt et al., 1994). A higher amount of aspartyl protease and carboxypeptidase could result in an increase in the generation of flavor precursors during fermentation, which could lead to changes in the flavor profiles of roasted cocoa beans (Janek et al., 2016; Scollo et al., 2020b).

Papain family cysteine protease (A0A061FQ64, A0A061E6K3), and subtilisin-like serine endopeptidase family protein (A0A061FAP5) were found in significantly higher abundance in the EET19 genotype. Papain-like cysteine proteases (PLCPs), including papain, chymopapain, ficin and bromelain, are cysteine proteases capable of hydrolyzing peptides and proteins composed of carboxyl groups of amino acid residues such as arginine, lysine and glycine (Sebastián et al., 2018).

The functional role of PLCPs in cocoa has been supported by recent findings from Purbaningrum et al. (2023), who demonstrated that enzymatic hydrolysis of cocoa beans using bromelain increases hydrophobic amino acids such as alanine, tyrosine, valine, leucine, phenylalanine, and glycine, as the main flavor precursors and desirable volatile compounds, such as pyrazines. Subtilisin-like serine endopeptidase family protein is a serine protease, which uses a serine residue in the active site, for protein degradation activity (Sebastián et al., 2018).

Glutathione S-transferase (GST) family protein and xylem serine proteinase 1 (A0A061FFU2) were up-regulated in EET19 compared to PA121. A previous study by Scollo et al. (2020a) also reported elevated levels of GST proteins in the IMC67 genotype, with one GST showing the second-highest fold change among the genotypes studied. The GST family comprises a diverse group of multifunctional enzymes that catalyze the conjugation of glutathione, a tripeptide, to a wide range of endogenous and xenobiotic compounds (Mohsenzadeh et al., 2011). Notably, GST proteins have been implicated in protein degradation processes during cocoa fermentation, playing a potential role in flavor precursor formation (Colonges et al., 2022b).

The proteins 2-oxoglutarate and Fe(II)-dependent oxygenase superfamily protein, asparagine synthetase [glutamine-hydrolyzing] (A0A061DI19) and Map3k delta-1 protein kinase (A0A061FQC7) were also up-regulated in EET19 compared only to the CCN51. Asparagine synthetase [glutamine-hydrolyzing] can produce asparagine from glutamine and plays a crucial role in the development of cocoa flavor during roasting (Voigt and Lieberei, 2014).

In comparison to the P7 genotype, EET19 exhibited elevated levels of E1 ubiquitin-activating enzyme (fragment) and subtilisin-like serine endopeptidase family protein, suggesting enhanced proteolytic processing and protein turnover that may support the generation of peptides and amino acids important for flavor development.

Interestingly, two entries of the alpha/beta-hydrolases superfamily protein A0A061GTX8 and A0A061E2W9 were up-regulated and down-regulated in EET19, respectively. Colonges et al. (2021) identified 100 candidate genes for the pyrazine association zones in National cocoa, of which 25 genes coding for the alpha/beta-hydrolases superfamily protein with peptidase function.

Cocoa flavor compounds are partially derived from the fatty acid degradation pathway, as previously reported (Colonges et al., 2022b). This pathway is responsible for the synthesis of many volatile straight-chain aldehydes, alcohols, ketones and esters, which originated from unsaturated fatty acids (linolenic acid and linoleic acid) as precursors via the lipoxygenase (LOX) pathway (El Hadi et al., 2013). Linoleic and linolenic acids are converted into the hydroperoxide intermediate catalyzed by LOX and then form corresponding straight-chain aldehydes, alcohols, and esters under the catalysis of hydroperoxide lyase, alcohol dehydrogenase, and alcohol acyltransferase (El Hadi et al., 2013).

Acyl carrier proteins (ACPs) are involved in the fatty acid pathway leading to volatile compound formation in many plants (Zhang et al., 2015). The methylketones and their corresponding secondary alcohols, which contribute fruity aroma notes, are considered to originate from fatty acid-derived metabolism. These ketones can be synthetized by hydrolysis and subsequent decarboxylation of β-ketoacyl-ACPs, catalyzed by methylketone synthase (El Hadi et al., 2013) The ketones 2-heptanone and 2-pentanone, found in fine flavor genotypes EET62 and SCA6 (Kadow et al., 2013), are likely precursors of the secondary alcohols 2-heptanol and 2-pentanol from many acyl ACPs. Besides, 2-hexanol may be formed as a downstream product of the LOX pathway, through the reduction of aldehydes such as hexanal (Rottiers et al., 2019).

GDSL-motif lipase/hydrolase is a lipase/esterase responsible for the formation of acetate esters such as benzyl acetate from benzyl alcohol or the synthesis of 1-phenyl acetate from 1-phenyl ethanol (Colonges et al., 2021). Esters are generally known as one of the most important contributors to the fruity and floral flavor notes of cocoa, synthesized from the esterification of higher alcohols obtained from previous pathways (Colonges et al., 2022a; Rottiers et al., 2019).

Previous studies focused on floral aromas of cocoa identified secondary metabolite biosynthetic pathways involved in fine flavor synthesis (Colonges et al., 2021; 2022a). Products of the cinnamoyl alcohol are associated with the production of flavor such as floral, cinnamon, and balsamic taste in the fruits (Pan et al., 2014). Alcohol dehydrogenase could catalyze aroma volatile by interconverting alcohols and aldehydes, which increase the fine flavors NADP-dependent alcohol dehydrogenase activities in strawberries were found to have broad substrate specificities including those alcohols and aldehydes responsible for strawberry aroma and flavor either directly or through their ester products (Mitchell and Jelenkovic, 1995). CAD and ADH in genotypes EET19 and P7 support their involvement in flavor development.

Non-volatile sugar conjugates represent an important reservoir of precursors for the biosynthesis of key flavor volatiles in plants (El Hadi et al., 2013). The synthesis and hydrolysis of these sugar conjugates are mediated by glycosyltransferases and glycosidases, respectively, which play a role in modulating the release and availability of volatile aroma in cocoa beans. In particular, family 1 glycosyltransferases (UGTs) catalyze the transfer of glycosyl groups from UDP-sugars to a diverse array of acceptor molecules, including terpenoids, phenolic compounds and other secondary metabolites. This influences the storage and release of flavor-active compounds (Liu et al., 2015). In sweet orange, for example, Fan et al. (2010) identified three putative terpenoid UGT genes involved in terpenoid glycosylation. Interestingly, UGTs are also involved in the biosynthesis of proanthocyanidins, indicating their broader role in secondary metabolism (Liu et al., 2015).

Chalcone-flavonone isomerase is involved in the flavonoid biosynthetic pathway, which isomerase the chalcone into flavanone (Meng et al., 2022). The enzyme benzoyl coenzyme A: Benzyl alcohol benzoyl transferase plays a role in the biosynthesis of volatile benzenoids and benzoic acid derivatives. This enzyme was described to catalyses the formation of 2-phenylethyl benzoate from benzoyl-CoA and 2-phenylethanol in *Petunia hybrida* (Boatright et al., 2004). The fruity and floral aroma notes in cocoa and other aromatic plants are significantly contributed to by these esters (Colonges et al., 2021; Kadow, 2020).

Volatile terpenoids including sesquiterpenoids, hemiterpenoids, monoterpenoids, and diterpenoids are essential contributors to fruit aroma. These compounds are primarily synthesized via the mevalonic acid and methylerythritol phosphate pathway, with terpene synthase being the most critical enzyme for the final synthesis of terpenes (El Hadi et al., 2013). Interestingly, active flavor compounds, including terpenes, are found in unfermented cocoa beans (Qin et al., 2017). The fine flavor National cocoa genotypes are renowned for their high concentrations of terpenoids (Scollo et al., 2020a). Contrary to expectations for the National-type cocoa, the genotype EET19 exhibited a relatively low abundance of proteins involved in terpene biosynthesis pathway such as (+)-delta-cadinene synthase, 3S-linalool/(E)-nerolidol/(E,E)-geranyl linalool synthase, 4-hydroxy-3-methylbut-2-enyl diphosphate synthase isoform 1, terpene cyclase/mutase family member and involved in the L-phenylalanine degradation pathway including alcohol dehydrogenase 1 and cinnamoyl-CoA reductase 1 isoform 1 compared to the others genotypes. In contrast, the highest abundance of proteins involved in the terpenoids biosynthesis was observed in the flavor genotype P7. Collin et al. (2023) reported a linalool concentration 20 times higher in P7 (297 µg/Kg) than in the Brazilian fine cocoa genotype Catongo. Furthermore, Kumari et al. (2018) found elevated levels of the 3S-linalool synthase protein in CCN51 cocoa from Africa, suggesting that even bulk genotypes may possess some aromatic potential under certain conditions. However, storage proteins, especially the 21 kDa albumin were found at high level in EET19 compared to the other genotypes. On the other hand, P7 and PA121 genotypes showed increased levels of enzymes involved in peptide and sugar degradation pathways, which are essential for Maillard reaction-derived volatiles, as well as those in the L-phenylalanine and fatty acid degradation pathways.

With regard to the main biosynthesis pathway involved in the synthesis of the floral and fruit aroma in the National cocoa, Colonges et al. (2021) identified different areas of association for monoterpene pathways in fermented roasted and unroasted cocoa beans. Additionally, De Wever (2020) demonstrated that, in fine flavor cocoa, the expression of genes from the secondary metabolite pathway including the terpenoid pathway is strongly upregulated during fermentation. This may elucidate the tenfold increase of the linalool concentration found in the cotyledon tissue as reported by Chetschik et al. (2018). Although terpenes have been found in unfermented National cultivars, the findings obtained in this study suggest that fine flavor compounds can be produced also in the cotyledons and are not always fruit-pulp derived, and highly increasing during fermentation.

## 5 Conclusion

In this study, more than 2,000 endogenous proteins were identified and quantified in unfermented cocoa beans from four genotypes. The four cocoa beans genotypes studied here exhibited distinct protein abundances, encompassing storage proteins, enzymes and structural proteins. The fine-flavor National cocoa genotype EET19 showed elevated levels of storage proteins, whereas the bulk CCN51 displayed a higher abundance of specific enzymes, notably those involved in lipid biosynthesis. In general, the formation of flavor precursors during fermentation is apparently not limited by the levels of enzymes in unfermented beans. The proteomic comparation of reference genotypes with contrasting organoleptic profiles, such as the fine-flavor Nacional cultivar and the bulk-type CCN51, provide insights into key proteins and their putative roles in fine flavor development. This study is valuable in contributing and deploying the information into public data repositories that can be used in the future for gene annotation, model predicting pathways and the design of targeted approaches.

## Data Availability

The datasets presented in this study can be found in online repositories. The names of the repository/repositories and accession number(s) can be found in the article/**Supplementary Material**.

## Supplementary material

Supplementary Table 1 The total identified proteins.

## References

[B1] Abid G. Jebara M. Debode F. Vertommen D. Ruys S. P. D. Ghouili E.. (2024). Comparative physiological, biochemical and proteomic analyses reveal key proteins and crucial regulatory pathways related to drought stress tolerance in faba bean (*Vicia faba* L.) leaves. Curr. Plant Biol. 37, 100320. doi: 10.1016/j.cpb.2024.100320

[B2] Afzaal M. Saeed F. Hussain M. Shahid F. Siddeeg A. Al-Farga A. (2022). Proteomics as a promising biomarker in food authentication, quality and safety: A review. Food Sci. Nutr. 10, 2333–2346. doi: 10.1002/fsn3.2842, PMID: 35844910 PMC9281926

[B3] Boatright J. Negre F. Chen X. Kish C. M. Wood B. Peel G.. (2004). Understanding *in vivo* benzenoid metabolism in petunia petal tissue. Plant Physiol. 135, 1993–2011. doi: 10.1104/pp.104.045468, PMID: 15286288 PMC520771

[B4] Boza E. J. Motamayor J. C. Amores F. M. Cedeño-Amador S. Tondo C. L. Livingstone D. S. III. (2014). Genetic characterization of the cacao cultivar CCN 51: Its impact and significance on global cacao improvement and production. J. Amer. Soc Hortic. Sci. 139, 219–229. doi: 10.21273/JASHS.139.2.219

[B5] Bradford M. M. (1976). A rapid and sensitive method for the quantitation of microgram quantities of protein utilizing the principle of protein-dye binding. Anal. Biochem. 72, 248–254. doi: 10.1016/0003-2697(76)90527-3, PMID: 942051

[B6] Chen C. He B. Liu X. Ma X. Liu Y. Yao H.-Y.. (2020). Pyrophosphate-fructose 6-phosphate 1-phosphotransferase (PFP1) regulates starch biosynthesis and seed development via heterotetramer formation in rice (*Oryza sativa* L.). Plant Biotechnol. J. 18, 83–95. doi: 10.1111/pbi.13173, PMID: 31131526 PMC6920184

[B7] Chetschik I. Kneubühl M. Chatelain K. Schlüter A. Bernath K. Hühn T. (2018). Investigations on the aroma of cocoa pulp (Theobroma cacao L.) and its influence on the odor of fermented cocoa beans. J. Agric. Food Chem. 66, 2467–2472. doi: 10.1021/acs.jafc.6b05008, PMID: 28318272

[B8] Collin S. Fisette T. Pinto A. Souza J. Rogez H. (2023). Discriminating aroma compounds in five cocoa bean genotypes from two Brazilian states: White Kerosene-like Catongo, Red Whisky-like FL89 (Bahia), Forasteros IMC67, PA121 and P7 (Pará). Mol 28, 1548. doi: 10.3390/molecules28041548, PMID: 36838536 PMC9961520

[B9] Colonges K. Jimenez J. C. Lahon M.-C. Seguine E. Calderon D. Subia C.. (2022b). Variability and genetic determinants of native cocoa trees aromas from South Ecuadorian Amazonia. Plant People Planet. 4, 618–637. doi: 10.1002/ppp3.10268

[B10] Colonges K. Jimenez J. C. Saltos A. Seguine E. Loor Solorzano R. G. Fouet O.. (2021). Two main biosynthesis pathways involved in the synthesis of the floral aroma of the Nacional cocoa variety. Front. Plant Sci. 12. doi: 10.3389/fpls.2021.681979, PMID: 34630447 PMC8498224

[B11] Colonges K. Jimenez J.-C. Saltos A. Seguine E. Loor Solorzano R. G. Fouet O.. (2022a). Integration of GWAS, metabolomics, and sensorial analyses to reveal novel metabolic pathways involved in cocoa fruity aroma GWAS of fruity aroma in *Theobroma cacao* . Plant Physiol. Biochem. 171, 213–225. doi: 10.1016/j.plaphy.2021.11.006, PMID: 34863583

[B12] Colonges K. Seguine E. Saltos A. Davrieux F. Minier J. Jimenez J.-C.. (2022c). Diversity and determinants of bitterness, astringency, and fat content in cultivated Nacional and native Amazonian cocoa accessions from Ecuador. Plant Genome 15, e20218. doi: 10.1002/tpg2.20218, PMID: 36065790 PMC12806967

[B13] Der Agopian R. G. Fabi J. P. Cordenunsi-Lysenko B. R. (2020). Metabolome and proteome of ethylene-treated papayas reveal different pathways to volatile compounds biosynthesis. Food Res. Int. 131, 108975. doi: 10.1016/j.foodres.2019.108975, PMID: 32247445

[B14] Deus V. L. Bispo E. Franca A. S. Gloria M. B. A. (2020). Influence of cocoa clones on the quality and functional properties of chocolate - nitrogenous compounds. LWT – Food Sci. Technol. 134, 1–7. doi: 10.1016/j.lwt.2020.110202

[B15] D'Souza R. N. Grimbs A. Grimbs S. Behrends B. Corno M. Ullrich M. S.. (2018). Degradation of cocoa proteins into oligopeptides during spontaneous fermentation of cocoa beans. Food Res Int. 109, 506–516. doi: 10.1016/j.foodres.2018.04.068, PMID: 29803477

[B16] De Wever J. (2020). Genotyping and bean transcriptomics – towards identification of cacao flavor genes. (PhD thesis). (Belgium: Ghent University), 285.

[B17] El Hadi M. A. Zhang F. J. Wu F. F. Zhou C. H. Tao J. (2013). Advances in fruit aroma volatile research. Mol 18, 8200–8229. doi: 10.3390/molecules18078200, PMID: 23852166 PMC6270112

[B18] Fan J. Chen C. Yu Q. Li Z. G. Gmitter F. G. Jr. (2010). Characterization of three terpenoid glycosyltransferase genes in ‘Valencia’ sweet orange (*Citrus sinensis* L. Osbeck). Genome 53, 816–823. doi: 10.1139/g10-068, PMID: 20962888

[B19] Hansen C. E. Mañez A. Burri C. Bousbaine A. (2000). Comparison of enzyme activities involved in flavour precursor formation in unfermented beans of different cocoa genotypes. J. Sci. Food Agric. 80, 1193–1198. doi: 10.1002/10970010(200006)80:8<1193::AIDJSFA619>3.0.CO;2-7

[B20] Herrera-Rocha F. Fernández-Niño M. Cala M. P. Duitama J. Barrios A. F. G. (2023). Omics approaches to understand cocoa processing and chocolate flavor development: A review. Food Res. Int. 165, 112555. doi: 10.1016/j.foodres.2023.112555, PMID: 36869541

[B21] Janek K. Niewienda A. Wöstemeyer J. Voigt J. (2016). The cleavage specificity of the aspartic protease of cocoa beans involved in the generation of the cocoa-specific aroma precursors. Food Chem. 211, 320–328. doi: 10.1016/j.foodchem.2016.05.033, PMID: 27283639

[B22] Kadow D. (2020). The biochemistry of cocoa flavor – A holistic analysis of its development along the processing chain. J. Appl. Bot. Food Qual. 93, 300–312. doi: 10.5073/JABFQ.2020.093.037

[B23] Kadow D. Bohlmann J. Phillips W. Lieberei R. (2013). Identification of main fine or flavour components in two genotypes of the cocoa tree (*Theobroma cacao* L.). J. Appl. Bot. Food Qual. 86, 90–98. doi: 10.5073/JABFQ.2013.086.013

[B24] Kumari N. Grimbs A. D’Souza R. N. Verma S. K. Corno M. Kuhnert N.. (2018). Origin and varietal based proteomic and peptidomic fingerprinting of *Theobroma cacao* in non-fermented and fermented cocoa beans. Food Res. Int. 111, 137–147. doi: 10.1016/j.foodres.2018.05.010, PMID: 30007670

[B25] Liu S. Kerr E. D. Pegg C. L. Schulz B. L. (2022). Proteomics and glycoproteomics of beer and wine. Proteomics 22, e2100329. doi: 10.1002/pmic.202100329, PMID: 35716130

[B26] Meng L. Zhang S. Bai X. Li X. Wang Q. Wang L.. (2022). Transcriptomic and Non-Targeted Metabolomic Analyses Reveal the Flavonoid Biosynthesis Pathway in Auricularia cornea. Molecules 27, 2334. doi: 10.3390/molecules27072334, PMID: 35408732 PMC9000485

[B27] Liu Y. Shi Z. Maximova S. N. Payne M. J. Guiltinan M. J. (2015). *Tc-MYBPA* is an Arabidopsis TT2-like transcription factor and functions in the regulation of proanthocyanidin synthesis in *Theobroma cacao* . BMC Plant Biol. 15, 160. doi: 10.1186/s12870-015-0529-y, PMID: 26109181 PMC4481123

[B28] Minges A. Ciupka D. Winkler C. Hoppner A. Gohlke H. Groth G. (2017). Structural intermediates and directionality of the swiveling motion of Pyruvate Phosphate Dikinase. Sci. Rep. 7, 45389. doi: 10.1038/srep45389, PMID: 28358005 PMC5371819

[B29] Mitchell W. C. Jelenkovic G. (1995). Characterizing NAD- and NADP-dependent alcohol dehydrogenase enzymes of strawberries. J. Am. Soc Hortic. Sci. 120, 798–801. doi: 10.21273/JASHS.120.5.798

[B30] Mohsenzadeh S. Esmaeili M. Moosavi F. Shahrtash M. Saffari B. Mohabatkar H. (2011). Plant glutathione S-transferase classification, structure and evolution. Afr. J. Biotechnol. 10, 8160–8165. doi: 10.5897/AJB11.1024

[B31] Pan H. Zhou R. Louie G. V. Mühlemann J. K. Bomati E. K. Bowman M. E. (2014). Structural studies of cinnamoyl-CoA reductase and cinnamyl-alcohol dehydrogenase, key enzymes of monolignol biosynthesis. Plant Cell 26, 3709–3727. doi: 10.1105/tpc.114.127399, PMID: 25217505 PMC4213152

[B32] Perez-Riverol Y. Bai J. Bandla C. García-Seisdedos D. Hewapathirana S. KamatChinathan S.. (2022). The PRIDE database resources in 2022: a hub for mass spectrometry-based proteomics evidences. Nucleic Acids Res. 50, D543–D552. doi: 10.1093/nar/gkab1038, PMID: 34723319 PMC8728295

[B33] Purbaningrum K. Hidayat C. Witasari L. D. Utami T. (2023). Flavor precursors and volatile compounds improvement of unfermented cocoa beans by hydrolysis using bromelain. Foods 12, 820. doi: 10.3390/foods12040820, PMID: 36832893 PMC9956981

[B34] Qin X.-W. Lai J.-X. Tan L.-H. Hao C.-Y. Li F.-P. He S.-Z.. (2017). Characterization of volatile compounds in Criollo, Forastero, and Trinitario cocoa seeds (*Theobroma cacao* L.) in China. Int. J. Food Prop. 20, 2261–2275. doi: 10.1080/10942912.2016.1236270

[B35] Rawel H. M. Huschek G. Sagu S. T. Homann T. (2019). Cocoa bean proteins-characterization, changes and modifications due to ripening and post-harvest processing. Nutrients 11, 428. doi: 10.3390/nu11020428, PMID: 30791360 PMC6413064

[B36] R Core Team. (2024). R: A Language and Environment for Statistical Computing. R Foundation for Statistical Computing, Vienna, Austria. Available online at: https://www.R-project.org/.

[B37] Rottiers H. Tzompa Sosa D. A. De Winne A. Ruales J. De Clippeleer J. De Leersnyder I.. (2019). Dynamics of volatile compounds and flavor precursors during spontaneous fermentation of fine flavor Trinitario cocoa beans. Eur. Food Res. Technol. 245, 1917–1937. doi: 10.1007/s00217-019-03307-y

[B38] Santander Muñoz M. Cortina J. R. Vaillant F. E. Parra E. S. (2019). An overview of the physical and biochemical transformation of cocoa seeds to beans and to chocolate: Flavor formation. Crit. Rev. Food Sci. Nutr. 60, 1593–1613. doi: 10.1080/10408398.2019.1581726, PMID: 30896305

[B39] Scalone G. L. L. Textoris-Taube K. Meulenaer B. Kimpe N. Wöstemeyer J. Voigt J. (2019). Cocoa-specific flavor components and their peptide precursors. Food Res. Int. 123, 503–515. doi: 10.1016/j.foodres.2019.05.019, PMID: 31284999

[B40] Scollo E. Neville D. Oruna-Concha M. J. Trotin M. Cramer R. (2018). Characterization of the proteome of *theobroma cacao* beans by nano-UHPLC-ESI MS/MS. Proteom 18, 3–4. doi: 10.1002/pmic.201700339, PMID: 29280563

[B41] Scollo E. Neville D. C. A. Oruna-Concha M. J. Trotin M. Cramer R. (2020a). UHPLC-MS/MS analysis of cocoa bean proteomes from four different genotypes. Food Chem. 303, 125244. doi: 10.1016/j.foodchem.2019.125244, PMID: 31445177

[B42] Scollo E. Neville D. C. A. Oruna-Concha M. J. Trotin M. Umaharan P. Sukha D.. (2020b). Proteomic and peptidomic UHPLC-ESI MS/MS analysis of cocoa beans fermented using the Styrofoam-box method. Food Chem. 316, 126350. doi: 10.1016/j.foodchem.2020.126350, PMID: 32045819

[B43] Sebastián D. Guevara M. G. Rocío T. F. Virginia T. C. (2018). An overview of plant proteolytic enzymes. In Biotechnological Applications of Plant Proteolytic Enzymes, Guevara M. Daleo G. (eds). (Cham: Springer). doi: 10.1007/978-3-319-97132-2_1

[B44] Voigt J. Biehl B. Kamaruddin S. Wazir S. (1993). The major seed proteins of *Theobroma cacao* L. Food Chem. 47, 145–151. doi: 10.1016/0308-8146(93)90236-9

[B45] Voigt J. Lieberei R. (2014). “Biochemistry of cocoa fermentation,” in Cocoa and coffee fermentations. Eds. Schwan R. F. Fleet G. H. (CRC Press, Boca Raton), 103–225.

[B46] Voigt J. Biehl B. Heinrichs H. Kamaruddin S. Gaim Marsoner G. Hugi A. (1994). In-vitro formation of cocoa-specific aroma precursors: aroma-related peptides generated from cocoa-seed protein by co-operation of an aspartic endoprotease and a carboxypeptidase. Food Chem. 49, 173–180. doi: 10.1016/0308-8146(94)90155-4

[B47] Wiśniewski J. Zougman A. Nagaraj N. Mann M. (2009). Universal sample preparation method for proteome analysis. Nat. Methods 6, 359–362. doi: 10.1038/nmeth.1322, PMID: 19377485

[B48] Zaman S. Shan Z. (2024). Literature review of proteomics approach associated with coffee. Foods 27, 13(11):1670. doi: 10.3390/foods13111670, PMID: 38890899 PMC11172319

[B49] Zhang Y. Maximova S. N. Guiltinan M. J. Hudson K. Drew G. N. (2015). Characterization of a stearoyl-acyl carrier protein desaturase gene family from chocolate tree, *Theobroma cacao* L. Front. Plant Sci. 6. doi: 10.3389/fpls.2015.00239, PMID: 25926841 PMC4396352

