# Supplementary material for: Comparative proteomic analysis of unfermented cocoa beans reveals key metabolic differences between fine-flavor and bulk genotypes
Source: Front Plant Sci. 2025 Oct 10;16:1674701. doi: 10.3389/fpls.2025.1674701 (PMC12549638; doi:10.3389/fpls.2025.1674701)
Supplement: Supplementary Table 1 — The total identified proteins. [file DataSheet1.docx]

**Comparative proteomic analysis of unfermented cocoa beans reveals key metabolic differences between fine-flavor and bulk genotypes**

Ana Caroline de Oliveira^1,2,^*, Didier Vertommen**^3^,** Sébastien Pyr dit Ruys^3,4^, Herve Rogez^5^, Frédéric Debode^1^, Dominique Mingeot^1^, Pierre Bertin^2^, Yordan Muhovski^1^**,***

^1^Department of Life Sciences, Unit Bioengineering, Walloon Agricultural Research Centre (CRA-W), Gembloux, Belgium

^2^Earth and Life Institute – Agronomy (ELI-a), Universite´ catholique de Louvain, Q5 Louvain-la-Neuve, Belgium

^3^MassProt Platform, de Duve Institute, Universite´ Catholique de Louvain Q6 (UCLouvain), Brussels, Belgium,

^4^Integrated Pharmacometrics, Pharmacogenomics and Pharmacokinetics Group (PMGK), Louvain Drug Research Institute (LDRI), Universite´ Catholique de Louvain (UCLouvain), Brussels, Belgium

^5^Centre for Valorisation of Amazonian Bioactive Compounds and Federal University of Para´ , Bele´ m, Para´ , Brazil

**Supplementary Material**

| a) 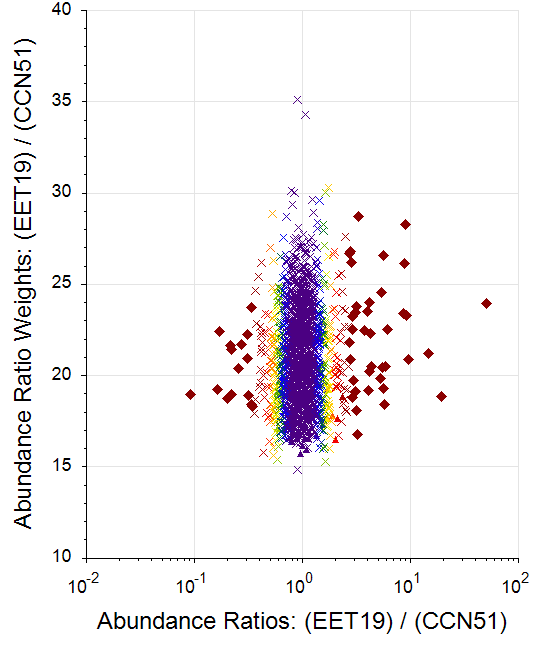 |
| --- |
| b) 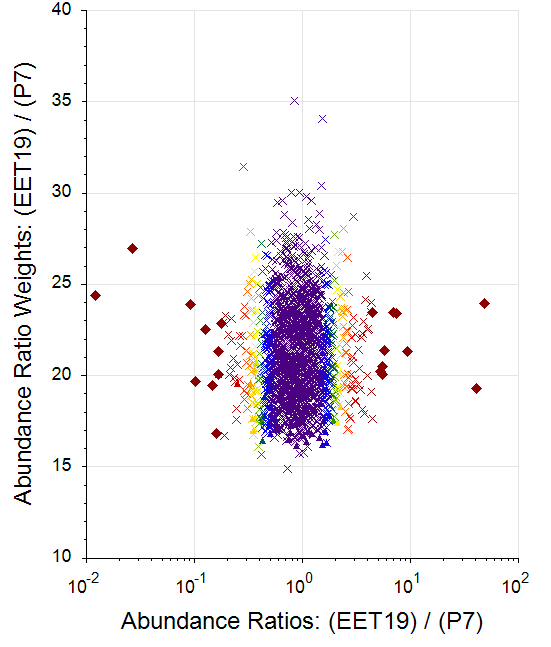 |
| c) 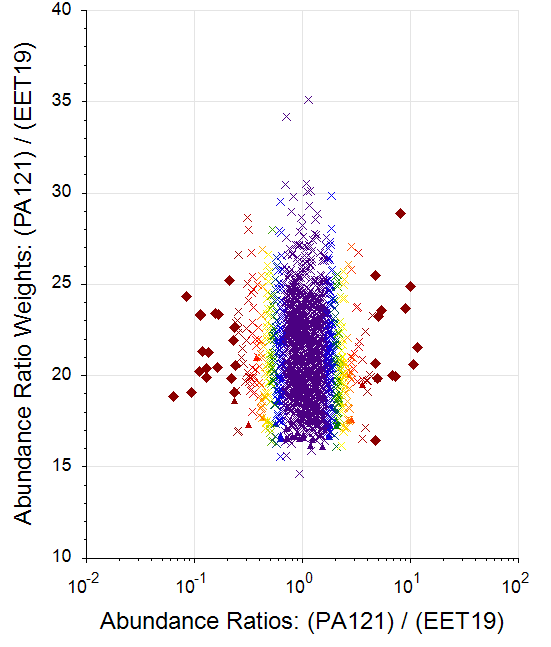 |

**Figure S1.** Graphical representation showing the cocoa genotypes proteome. A) Abundance ration weights vs. abundance ration plot of EET19 compared with CCN51 cocoa genotype. B) Abundance ration weights vs. abundance ration plot of EET19 compared with P7 cocoa genotype. C) Abundance ration weights vs. abundance ration plot of EET19 compared with PA121 cocoa genotype. Each plot represents the abundance of a protein, colored by the significance of the difference between two genotypes ranging from adj. p-value equal 1 (purple) to 6.26 x 10^-9^ (dark red). Diamond points represent the high confidence values (adj. p-value ≤ 0.001) and x-cross points the low confidence values (adj. p-value > 0.001). A threshold 2-fold increase (≥ 2.0) for up-regulated and 2-fold decrease (≤ 0.5) for down-regulated was applied. The up- and down-regulated significant protein are plotted at the right and left, respectively.

| A)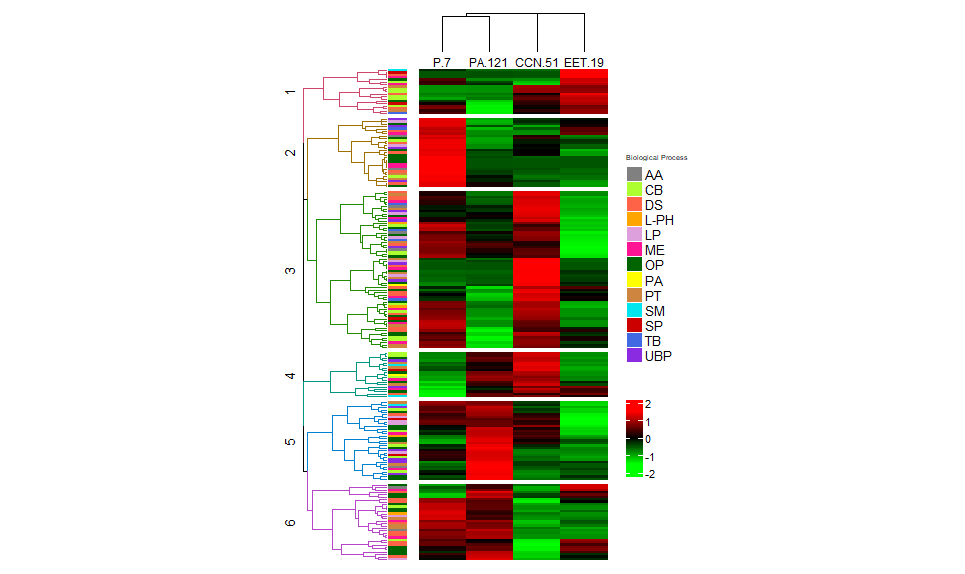 | B)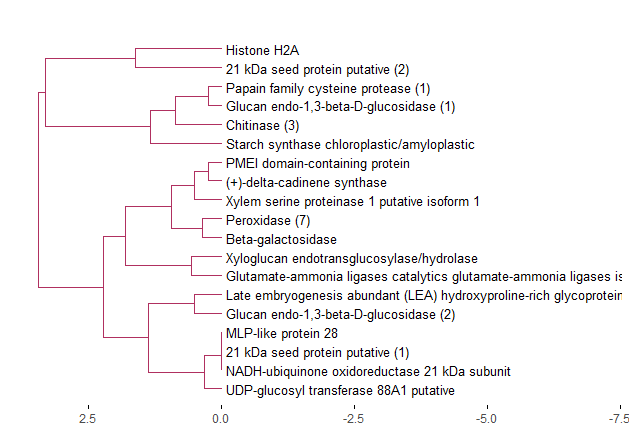  C)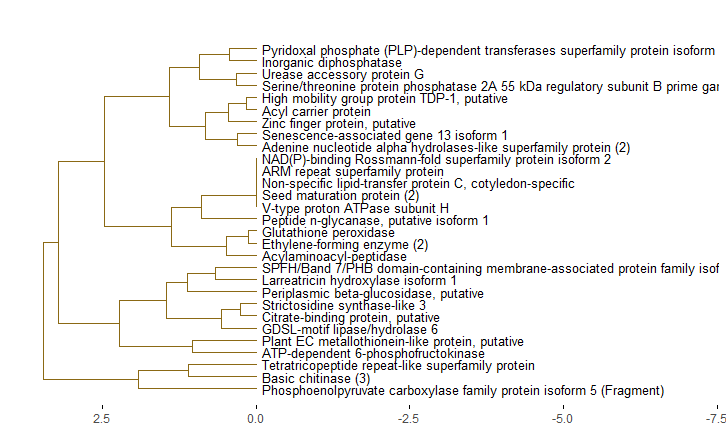 | D)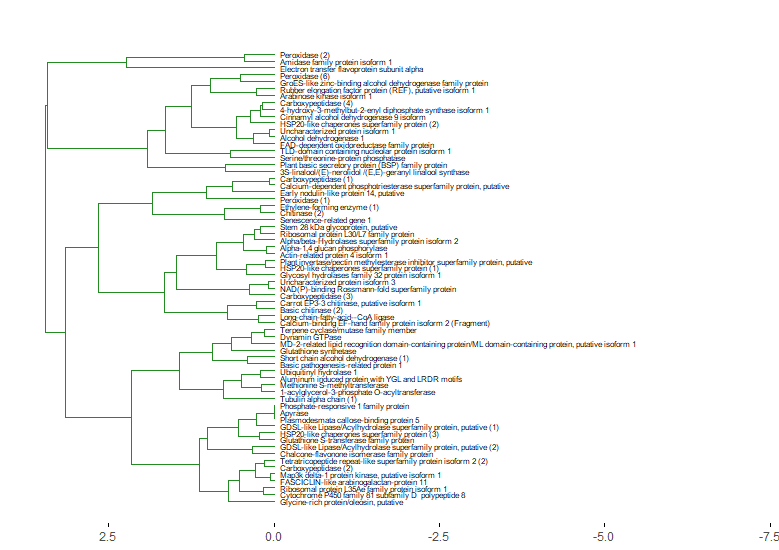 |
| --- | --- | --- |
| E)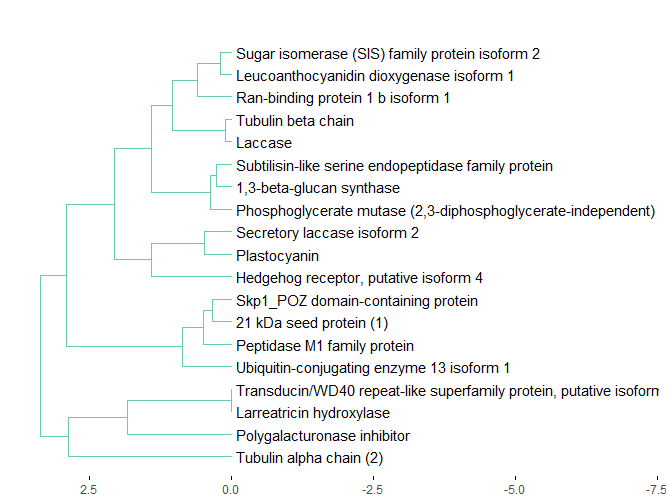 | F)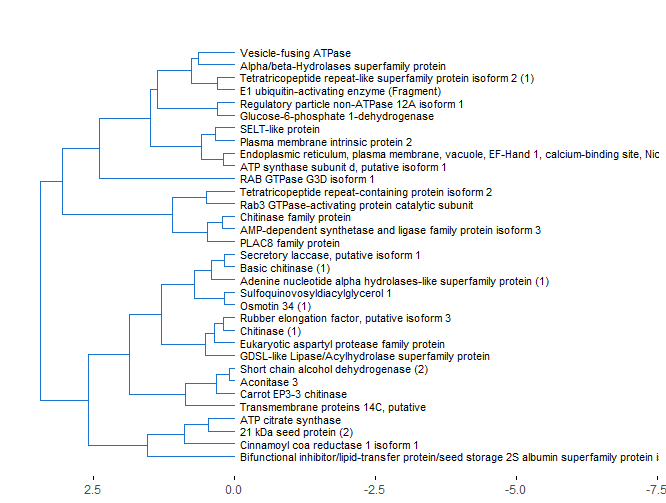 | G)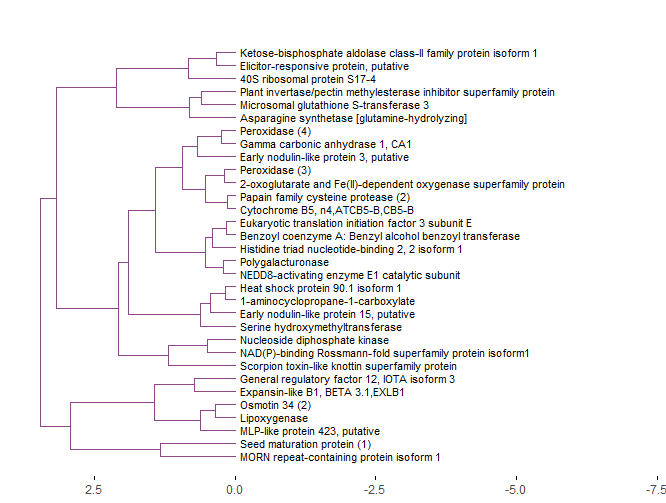 |

**Figure S2.** Hierarchical Cluster Analysis (HCA) representing the proteins with significant different abundances across cocoa genotypes from the proteomic analysis. (A) Heatmap showing six principal clusters of proteins. (B–G) Dendrogram representing clusters 1 to 6 from the HCA analysis, respectively. Each protein in the HCA is color coded based on their major biological process: Amino acid biosynthesis (AA), Carbohydrate metabolic process (CB), Defense and stress (DS), L-phenylalanine degradation (L-PH), Lipid metabolic process (LP), Metabolism and energy (ME), Others metabolic processes (OP), Proanthocyanin biosynthesis (PA), Protein metabolic process (PT), Secondary metabolism (SM), Storage protein (SP), Terpenoid biosynthesis (TB), Unspecified biological process (UBP).”

Table S6. Proteins from clusters 1 to 6 with significantly different abundances among the cocoa genotypes shown in the dendrograms obtained by hierarchical cluster analysis.

| **Proteins** | **Abundances** | | | |
| --- | --- | --- | --- | --- |
|  | **EET-19** | **CCN-51** | **P-7** | **PA-121** |
| **Cluster 1** |  |  |  |  |
| **Histone H2A** | 250,3 | 0 | 149,7 | 0 |
| **21 kDa seed protein, putative (2)** | 157,2 | 119,5 | 123,2 | 0 |
| **Papain family cysteine protease (1)** | 355,9 | 44,1 | 0 | 0 |
| **Glucan endo-1,3-beta-D-glucosidase (1)** | 395,8 | 4,2 | 0 | 0 |
| **Chitinase (3)** | 255,9 | 144,1 | 0 | 0 |
| **Starch synthase, chloroplastic/amyloplastic** | 221,3 | 178,7 | 0 | 0 |
| **PMEI domain-containing protein** | 54,3 | 202 | 143,7 | 0 |
| **(+)-delta-cadinene synthase** | 9,6 | 32,3 | 342,2 | 15,9 |
| **Xylem serine proteinase 1, putative isoform 1** | 87,5 | 287 | 25,5 | 0 |
| **Peroxidase (7)** | 98,5 | 241,5 | 60 |  |
| **Beta-galactosidase** | 84,6 | 266,7 | 48,7 | 0 |
| **Xyloglucan endotransglucosylase/hydrolase** | 67,2 | 0 | 191,9 | 140,9 |
| **Glutamate-ammonia ligases,catalytics,glutamate-ammonia ligases isoform 3** | 54 | 0 | 196,4 | 149,5 |
| **Late embryogenesis abundant (LEA) hydroxyproline-rich glycoprotein family, putative** | 273,3 | 126,7 | 0 | 0 |
| **Glucan endo-1,3-beta-D-glucosidase (2)** | 90,6 | 34,8 | 129,7 | 144,9 |
| **MLP-like protein 28** | 400 | 0 | 0 | 0 |
| **21 kDa seed protein, putative (1)** | 400 | 0 | 0 | 0 |
| **NADH-ubiquinone oxidoreductase 21 kDa subunit** | 400 | 0 | 0 | 0 |
| **UDP-glucosyl transferase 88A1, putative** | 277,5 | 0 | 122,5 | 0 |
| **Cluster 2** |  |  |  |  |
| **Pyridoxal phosphate (PLP)-dependent transferases superfamily protein isoform 1** | 0 | 142,5 | 257,5 | 0 |
| **Inorganic diphosphatase** | 21,6 | 36,9 | 95,1 | 246,4 |
| **Urease accessory protein G** | 0 | 179,7 | 88,8 | 131,5 |
| **Serine/threonine protein phosphatase 2A 55 kDa regulatory subunit B prime gamma, putative isoform 2** | 0 | 105,7 | 187 | 107,3 |
| **High mobility group protein TDP-1, putative** | 161,5 | 34,7 | 96,5 | 107,4 |
| **Acyl carrier protein** | 138,7 | 167,8 | 93,5 | 0 |
| **Zinc finger protein, putative** | 178,1 | 145,7 | 76,3 | 0 |
| **Senescence-associated gene 13 isoform 1** | 241,2 | 26,1 | 99,5 | 33,2 |
| **Adenine nucleotide alpha hydrolases-like superfamily protein (2)** | 145,1 | 155,1 | 99,7 | 0 |
| **NAD(P)-binding Rossmann-fold superfamily protein isoform 2** | 0 | 0 | 400 | 0 |
| **ARM repeat superfamily protein** | 0 | 0 | 400 | 0 |
| **Non-specific lipid-transfer protein C, cotyledon-specific** | 0 | 0 | 400 | 0 |
| **Seed maturation protein (2)** | 189,7 | 140,3 | 54,1 | 15,8 |
| **V-type proton ATPase subunit H** | 0 | 0 | 400 | 0 |
| **Peptide n-glycanase, putative isoform 1** | 0 | 0 | 289,6 | 110,4 |
| **Glutathione peroxidase** | 0 | 103,4 | 187,8 | 108,7 |
| **Ethylene-forming enzyme (2)** | 0 | 124 | 81,6 | 194,4 |
| **Acylaminoacyl-peptidase** | 18 | 139 | 69,5 | 173,5 |
| **SPFH/Band 7/PHB domain-containing membrane-associated protein family isoform 1** | 194,8 | 132 | 73,3 | 0 |
| **Larreatricin hydroxylase isoform 1** | 57,3 | 17,6 | 97 | 228,1 |
| **Periplasmic beta-glucosidase, putative** | 181,7 | 148,1 | 70,2 | 0 |
| **Strictosidine synthase-like 3** | 57,9 | 145,7 | 81,6 | 114,8 |
| **Citrate-binding protein, putative** | 179,4 | 12,2 | 208,4 | 0 |
| **GDSL-motif lipase/hydrolase 6** | 208,9 | 142,4 | 48,7 | 0 |
| **Plant EC metallothionein-like protein, putative** | 390,5 | 0 | 9,5 | 0 |
| **ATP-dependent 6-phosphofructokinase** | 207,4 | 0 | 192,6 | 0 |
| **Tetratricopeptide repeat-like superfamily protein** | 110,9 | 12,1 | 154,1 | 123 |
| **Basic chitinase (3)** | 100,8 | 10,5 | 167,2 | 121,5 |
| **Phosphoenolpyruvate carboxylase family protein isoform 5 (Fragment)** | 0 | 123,1 | 162,5 | 114,4 |
| **Cluster 3** |  |  |  |  |
| **Peroxidase (2)** | 139,8 | 134,7 | 125,4 | 0 |
| **Amidase family protein isoform 1** | 143,7 | 126,9 | 129,4 | 0 |
| **Electron transfer flavoprotein subunit alpha** | 22,2 | 199,7 | 162,4 | 15,8 |
| **Peroxidase (6)** | 213,3 | 50,4 | 79,5 | 56,8 |
| **GroES-like zinc-binding alcohol dehydrogenase family protein** | 0 | 128,3 | 148,2 | 123,6 |
| **Rubber elongation factor protein (REF), putative isoform 1** | 0 | 19,6 | 241,5 | 138,9 |
| **Arabinose kinase isoform 1** | 0 | 16,2 | 248,6 | 135,2 |
| **Carboxypeptidase (4)** | 0 | 33,9 | 77,9 | 288,2 |
| **4-hydroxy-3-methylbut-2-enyl diphosphate synthase isoform 1** | 0 | 25,5 | 220,4 | 154,2 |
| **Cinnamyl alcohol dehydrogenase 9 isoform** | 267,9 | 53,7 | 53,3 | 25,2 |
| **HSP20-like chaperones superfamily protein (2)** | 0 | 149,5 | 133,5 | 117 |
| **Uncharacterized protein isoform 1** | 176,5 | 64,7 | 93,6 | 65,2 |
| **Alcohol dehydrogenase 1** | 29,5 | 62 | 53,5 | 255 |
| **FAD-dependent oxidoreductase family protein** | 0 | 54,1 | 68,3 | 277,5 |
| **TLD-domain containing nucleolar protein isoform 1** | 0 | 149,7 | 128,8 | 121,6 |
| **Serine/threonine-protein phosphatase** | 0 | 135,7 | 133,2 | 131,1 |
| **Plant basic secretory protein (BSP) family protein** | 301,1 | 46,4 | 27,8 | 24,6 |
| **3S-linalool/(E)-nerolidol /(E,E)-geranyl linalool synthase** | 23,2 | 32,3 | 322,4 | 22,1 |
| **Carboxypeptidase (1)** | 255,2 | 63,8 | 81 | 0 |
| **Calcium-dependent phosphotriesterase superfamily protein, putative** | 199,5 | 52,6 | 45 | 102,9 |
| **Early nodulin-like protein 14, putative** | 265,5 | 43,3 | 91,2 | 0 |
| **Peroxidase (1)** | 317,7 | 33 | 49,3 | 0 |
| **Ethylene-forming enzyme (1)** | 21,4 | 54,2 | 218 | 106,5 |
| **Chitinase (2)** | 90,3 | 96,2 | 80,1 | 133,5 |
| **Senescence-related gene 1** | 93,5 | 98,7 | 207,8 | 0 |
| **Stem 28 kDa glycoprotein, putative** | 28,5 | 95,5 | 97,1 | 178,9 |
| **Ribosomal protein L30/L7 family protein** | 47,4 | 72,4 | 54,2 | 226 |
| **Alpha/beta-Hydrolases superfamily protein isoform 2** | 8,9 | 91,3 | 91,4 | 208,4 |
| **Alpha-1,4 glucan phosphorylase** | 0 | 61,2 | 338,8 | 0 |
| **Actin-related protein 4 isoform 1** | 0 | 235,9 | 164,1 | 0 |
| **Plant invertase/pectin methylesterase inhibitor superfamily protein, putative** | 0 | 211 | 189 | 0 |
| **HSP20-like chaperones superfamily protein (1)** | 0 | 212,6 | 187,4 | 0 |
| **Glycosyl hydrolases family 32 protein isoform 1** | 0 | 34,8 | 245,1 | 120,1 |
| **Uncharacterized protein isoform 3** | 0 | 179,6 | 220,4 | 0 |
| **NAD(P)-binding Rossmann-fold superfamily protein** | 0 | 169,3 | 230,7 | 0 |
| **Carboxypeptidase (3)** | 140,4 | 48,1 | 88,9 | 122,6 |
| **Carrot EP3-3 chitinase, putative isoform 1** | 190,5 | 72,7 | 87,9 | 48,8 |
| **Basic chitinase (2)** | 185,5 | 81 | 98,4 | 35,1 |
| **Long-chain-fatty-acid--CoA ligase** | 0 | 81,9 | 87,5 | 230,6 |
| **Calcium-binding EF-hand family protein isoform 2 (Fragment)** | 0 | 94 | 84,3 | 221,7 |
| **Terpene cyclase/mutase family member** | 0 | 69,4 | 198,5 | 132,1 |
| **Dynamin GTPase** | 0 | 73,8 | 202,3 | 123,9 |
| **MD-2-related lipid recognition domain-containing protein / ML domain-containing protein, putative isoform 1** | 32,2 | 9,3 | 81,2 | 277,4 |
| **Glutathione synthetase** | 0 | 96,6 | 195,7 | 107,8 |
| **Short chain alcohol dehydrogenase (1)** | 10,9 | 99,3 | 265,1 | 24,7 |
| **Basic pathogenesis-related protein 1** | 132,9 | 52,6 | 199,9 | 14,7 |
| **Ubiquitinyl hydrolase 1** | 0 | 91,5 | 160,1 | 148,4 |
| **Aluminum induced protein with YGL and LRDR motifs** | 0 | 81,3 | 171 | 147,6 |
| **Methionine S-methyltransferase** | 0 | 80,5 | 161,5 | 158 |
| **1-acylglycerol-3-phosphate O-acyltransferase** | 0 | 72,9 | 149,8 | 177,2 |
| **Tubulin alpha chain (1)** | 0 | 61,6 | 150,8 | 187,6 |
| **Phosphate-responsive 1 family protein** | 0 | 400 | 0 | 0 |
| **Apyrase** | 0 | 400 | 0 | 0 |
| **Plasmodesmata callose-binding protein 5** | 0 | 400 | 0 | 0 |
| **GDSL-like Lipase/Acylhydrolase superfamily protein, putative (1)** | 107,1 | 292,9 | 0 | 0 |
| **HSP20-like chaperones superfamily protein (3)** | 183,7 | 73,2 | 143,1 | 0 |
| **Glutathione S-transferase family protein** | 104,4 | 181,3 | 114,3 | 0 |
| **GDSL-like Lipase/Acylhydrolase superfamily protein, putative (2)** | 120,5 | 38,1 | 119,3 | 122,1 |
| **Chalcone-flavonone isomerase family protein** | 177,5 | 88,3 | 0 | 134,2 |
| **Tetratricopeptide repeat-like superfamily protein isoform 2 (2)** | 108,6 | 190,4 | 0 | 101 |
| **Carboxypeptidase (2)** | 126,7 | 273,3 | 0 | 0 |
| **Map3k delta-1 protein kinase, putative isoform 1** | 137,5 | 41,8 | 108,4 | 112,3 |
| **FASCICLIN-like arabinogalactan-protein 11** | 189,2 | 91,8 | 0 | 118,9 |
| **Ribosomal protein L35Ae family protein isoform 1** | 129,3 | 270,7 | 0 | 0 |
| **Cytochrome P450, family 81, subfamily D, polypeptide 8, putative** | 27,9 | 63,6 | 174,3 | 134,2 |
| **Glycine-rich protein / oleosin, putative** | 29,2 | 65,2 | 157,5 | 148,1 |
| **Cluster 4** |  |  |  |  |
| **Sugar isomerase (SIS) family protein isoform 2** | 136,3 | 69,1 | 102,1 | 92,5 |
| **Leucoanthocyanidin dioxygenase isoform 1** | 0 | 60,4 | 0 | 339,6 |
| **Ran-binding protein 1 b isoform 1** | 0 | 222,7 | 0 | 177,3 |
| **Tubulin beta chain** | 0 | 49 | 112,8 | 238,2 |
| **Laccase** | 330,3 | 7,3 | 27,6 | 34,8 |
| **Subtilisin-like serine endopeptidase family protein** | 261 | 92,1 | 14 | 32,9 |
| **1,3-beta-glucan synthase** | 0 | 66,1 | 110,2 | 223,7 |
| **Phosphoglycerate mutase (2,3-diphosphoglycerate-independent)** | 0 | 69,3 | 102,8 | 227,9 |
| **Secretory laccase isoform 2** | 309,2 | 55,8 | 0 | 35 |
| **Plastocyanin** | 107,4 | 35,1 | 0 | 257,4 |
| **Hedgehog receptor, putative isoform 4** | 61,3 | 77,7 | 0 | 261 |
| **Skp1_POZ domain-containing protein** | 104,7 | 25 | 102,2 | 168,1 |
| **21 kDa seed protein (1)** | 0 | 81,2 | 134,1 | 184,6 |
| **Peptidase M1 family protein** | 0 | 82,9 | 127 | 190,1 |
| **Ubiquitin-conjugating enzyme 13 isoform 1** | 0 | 67,1 | 130,2 | 202,8 |
| **Transducin/WD40 repeat-like superfamily protein, putative isoform 2** | 147,6 | 133,8 | 0 | 118,6 |
| **Larreatricin hydroxylase** | 68,5 | 4,8 | 114,7 | 211,9 |
| **Polygalacturonase inhibitor** | 352,6 | 22,3 | 0 | 25,1 |
| **Tubulin alpha chain (2)** | 21,9 | 31,9 | 128,4 | 217,8 |
| **Cluster 5** |  |  |  |  |
| **Vesicle-fusing ATPase** | 0 | 215,9 | 106,6 | 77,5 |
| **Alpha/beta-Hydrolases superfamily protein** | 181,7 | 28,5 | 112,4 | 77,4 |
| **Tetratricopeptide repeat-like superfamily protein isoform 2 (1)** | 178,1 | 127,8 | 0 | 94,1 |
| **E1 ubiquitin-activating enzyme (Fragment)** | 102,3 | 108,5 | 0 | 189,3 |
| **Glucose-6-phosphate 1-dehydrogenase** | 0 | 148,5 | 152 | 99,6 |
| **SELT-like protein** | 0 | 0 | 0 | 400 |
| **Regulatory particle non-ATPase 12A isoform 1** | 0 | 109,6 | 117,1 | 173,3 |
| **Plasma membrane intrinsic protein 2** | 2,7 | 143,4 | 168,5 | 85,5 |
| **Endoplasmic reticulum, plasma membrane, vacuole, EF-Hand 1, calcium-binding site, Nicastrin isoform 2** | 174,6 | 0 | 125,5 | 99,9 |
| **ATP synthase subunit d, putative isoform 1** | 110 | 0 | 0 | 290 |
| **RAB GTPase G3D isoform 1** | 121,8 | 123,3 | 0 | 154,9 |
| **Tetratricopeptide repeat-containing protein isoform 2** | 0 | 105,8 | 129 | 165,2 |
| **Rab3 GTPase-activating protein catalytic subunit** | 0 | 0 | 142,9 | 257,1 |
| **Chitinase family protein** | 221,4 | 151,2 | 20,6 | 6,8 |
| **AMP-dependent synthetase and ligase family protein isoform 3** | 0 | 100,1 | 205,5 | 94,4 |
| **PLAC8 family protein** | 138,7 | 0 | 162,5 | 98,8 |
| **Secretory laccase, putative isoform 1** | 223,8 | 36,8 | 74,6 | 64,8 |
| **Basic chitinase (1)** | 180,5 | 152,1 | 24,9 | 42,5 |
| **Adenine nucleotide alpha hydrolases-like superfamily protein (1)** | 0 | 110,4 | 137,2 | 152,5 |
| **Sulfoquinovosyldiacylglycerol 1** | 197,8 | 56,9 | 75,7 | 69,6 |
| **Osmotin 34 (1)** | 237,7 | 50,2 | 47,6 | 64,5 |
| **Rubber elongation factor, putative isoform 3** | 0 | 115,1 | 137,3 | 147,6 |
| **Chitinase (1)** | 268 | 33,9 | 43,7 | 54,3 |
| **Eukaryotic aspartyl protease family protein** | 0 | 115,4 | 143 | 141,6 |
| **GDSL-like Lipase/Acylhydrolase superfamily protein** | 17 | 315,4 | 35,5 | 32 |
| **Short chain alcohol dehydrogenase (2)** | 0 | 235,2 | 160,3 | 4,5 |
| **Aconitase 3** | 0 | 89,5 | 250,4 | 60,1 |
| **Carrot EP3-3 chitinase** | 163,5 | 37 | 192,7 | 6,7 |
| **Transmembrane proteins 14C, putative** | 0 | 124,5 | 126,2 | 149,3 |
| **ATP citrate synthase** | 0 | 127,6 | 130,2 | 142,3 |
| **21 kDa seed protein (2)** | 250,1 | 55,1 | 51 | 43,7 |
| **Cinnamoyl coa reductase 1 isoform 1** | 32,5 | 49,3 | 269,3 | 48,9 |
| **Bifunctional inhibitor/lipid-transfer protein/seed storage 2S albumin superfamily protein isoform 1** | 32 | 298 | 32,6 | 37,4 |
| **Cluster 6** |  |  |  |  |
| **Ketose-bisphosphate aldolase class-II family protein isoform 1** | 33,7 | 0 | 199,9 | 166,4 |
| **Elicitor-responsive protein, putative** | 6,2 | 125,1 | 32,4 | 236,3 |
| **40S ribosomal protein S17-4** | 150,9 | 0 | 124,9 | 124,2 |
| **Plant invertase/pectin methylesterase inhibitor superfamily protein** | 8,3 | 114,8 | 244,8 | 32,1 |
| **Microsomal glutathione S-transferase 3** | 41,2 | 0 | 122,4 | 236,4 |
| **Asparagine synthetase [glutamine-hydrolyzing]** | 73,2 | 0 | 128,6 | 198,3 |
| **Peroxidase (4)** | 191 | 114,9 | 60,9 | 33,2 |
| **Gamma carbonic anhydrase 1, CA1** | 0 | 0 | 226,3 | 173,7 |
| **Early nodulin-like protein 3, putative** | 220,1 | 103,7 | 45,3 | 30,9 |
| **Peroxidase (3)** | 319,2 | 15,4 | 36,9 | 28,6 |
| **2-oxoglutarate and Fe(II)-dependent oxygenase superfamily protein** | 107,1 | 0 | 149,5 | 143,5 |
| **Papain family cysteine protease (2)** | 157,8 | 116,3 | 98,4 | 27,5 |
| **Cytochrome B5, n4,ATCB5-B,CB5-B** | 0 | 0 | 241,8 | 158,2 |
| **Eukaryotic translation initiation factor 3 subunit E** | 192,2 | 133,9 | 47,6 | 26,3 |
| **Benzoyl coenzyme A: Benzyl alcohol benzoyl transferase** | 0 | 109,4 | 80,4 | 210,2 |
| **Histidine triad nucleotide-binding 2, 2 isoform 1** | 287,6 | 20,8 | 61,3 | 30,3 |
| **Polygalacturonase** | 30,9 | 264,2 | 67,1 | 37,8 |
| **NEDD8-activating enzyme E1 catalytic subunit** | 0 | 106,9 | 149,1 | 143,9 |
| **Heat shock protein 90.1 isoform 1** | 0 | 0 | 353,8 | 46,2 |
| **1-aminocyclopropane-1-carboxylate** | 0 | 0 | 186,6 | 213,4 |
| **Early nodulin-like protein 15, putative** | 148,6 | 127,9 | 60,3 | 63,2 |
| **Serine hydroxymethyltransferase** | 0 | 0 | 311,3 | 88,7 |
| **Nucleoside diphosphate kinase** | 162,7 | 0 | 16,6 | 220,7 |
| **NAD(P)-binding Rossmann-fold superfamily protein isoform 1** | 18 | 0 | 342,8 | 39,2 |
| **Scorpion toxin-like knottin superfamily protein** | 42,5 | 0 | 43,2 | 314,3 |
| **General regulatory factor 12, IOTA isoform 3** | 57,1 | 168,8 | 124,2 | 49,9 |
| **Expansin-like B1, BETA 3.1,EXLB1** | 66,8 | 18,5 | 255,9 | 58,8 |
| **Osmotin 34 (2)** | 291,9 | 13,2 | 13,3 | 81,5 |
| **Lipoxygenase** | 34,9 | 23,4 | 283,5 | 58,2 |
| **MLP-like protein 423, putative** | 343,7 | 17,9 | 31,5 | 6,9 |
| **Seed maturation protein (1)** | 250,6 | 12,6 | 121,3 | 15,5 |
| **MORN repeat-containing protein isoform 1** | 145,1 | 113,5 | 0 | 141,4 |
