# Supplementary material for: Comparative proteomic analysis of unfermented cocoa beans reveals key metabolic differences between fine-flavor and bulk genotypes
Source: Front Plant Sci. 2025 Oct 10;16:1674701. doi: 10.3389/fpls.2025.1674701 (PMC12549638; doi:10.3389/fpls.2025.1674701)
Supplement: Supplementary file 3 [file Table2.docx]

**Table S5.** Differentially abundant protein (DAPs) from the four cocoa genotypes analyzed by LC-MS/MS.

| **Accession** | **Protein Name** | **Molecular Function** | **Regulated Type** | | | **Peptides/ Coverage (%)** | **MW [kDa]** | **calc. pI** | **Abundance ratios*** | | |
| --- | --- | --- | --- | --- | --- | --- | --- | --- | --- | --- | --- |
|  |  |  | **EET-19/**  **CCN-51** | **EET-19/**  **P-7** | **EET-19/**  **PA-121** |  |  |  | **(EET-19)/**  **(CCN-51)** | **(EET-19) / (P-7)** | **(EET-19/**  **PA- 121)** |
| **Storage Protein** | |  |  |  |  |  |  |  |  |  |  |
| A0A061E8U0 | Stem 28 kDa glycoprotein, putative | Nutrient reservoir activity | ns | ns | ↓Down | 8 (35) | 29.1 | 9.13 | 0.41 | 0.35 | 0.19 |
| A0A061EYL9 | Bifunctional inhibitor/lipid-transfer protein/seed storage 2S albumin superfamily protein isoform 1 | Protease inhibitor/seed protein/lipid transfer | ↓Down | ns | ns | 3 (46) | 12.2 | 8.24 | 0.22 | 1.26 | 0.63 |
| A0A061FVK5 | 21 kDa seed protein | Endopeptidase inhibitor activity | ↓Down | ↓Down | ↓Down | 24 (76) | 23.9 | 6.39 | 0.01 | 0.01 | 0.01 |
| A0A061FWL5 | 21 kDa seed protein | Endopeptidase inhibitor activity | ↑Up | ns | ns | 25 (76) | 23.9 | 6.13 | 3.34 | 2.97 | 3.23 |
| A0A061E5P2 | 21 kDa seed protein, putative | Endopeptidase inhibitor activity | ↑Up | ↑Up | ↑Up | 2 (11) | 23.7 | 4.83 | 100.00 | 100.00 | 100.00 |
| A0A061G2L0 | 21 kDa seed protein, putative | Endopeptidase inhibitor activity | ns | ns | ↑Up | 1 (6) | 24.1 | 6.19 | 1.31 | 1.20 | 100.00 |
|  |  |  |  |  |  |  |  |  |  |  |  |
| **Protein metabolic process and amino acid biosynthesis** | |  |  |  |  |  |  |  |  |  |  |
| A0A061GYI1 | E1 ubiquitin-activating enzyme (Fragment) | Ubiquitin activating enzyme activity | ns | ↑Up | ns | 7 (12) | 115.6 | 5.29 | 0.96 | 100.00 | 0.56 |
| A0A061FQ64 | Papain family cysteine protease | Cysteine-type endopeptidase activity | ns | ↑Up | ↑Up | 3 (15) | 42.2 | 7.3 | 0.75 | 100.00 | 100.00 |
| A0A061E6K3 | Papain family cysteine protease | Cysteine-type endopeptidase activity | ns | ns | ↑Up | 2 (10) | 43.7 | 5.88 | 1.47 | 1.78 | 4.18 |
| A0A061FAP5 | Subtilisin-like serine endopeptidase family protein | Serine-type endopeptidase activity | ns | ↑Up | ns | 4 (9) | 81.7 | 5.55 | 2.06 | 5.35 | 2.72 |
| A0A061GEJ2 | Glutathione S-transferase family protein | Glutathione transferase activity | ns | ns | ↑Up | 6 (23) | 40.4 | 7.91 | 0.58 | 0.91 | 100.00 |
| A0A061EFD5 | Carboxypeptidase | Serine-type carboxypeptidase activity | ns | ns | ↑Up | 5 (14) | 53.3 | 8.81 | 2.47 | 1.95 | 100.00 |
| A0A061GQ08 | Carboxypeptidase | Serine-type carboxypeptidase activity | ns | ↑Up | ↑Up | 3 (8) | 53.3 | 5.97 | 0.90 | 100.00 | 100.00 |
| A0A061FIY5 | Carboxypeptidase | Serine-type carboxypeptidase activity | ↑Up | ns | ns | 5 (16) | 53 | 6.4 | 2.91 | 1.56 | 1.10 |
| A0A061H0B2 | Carboxypeptidase | Serine-type carboxypeptidase activity | ↓Down | ↓Down | ↓Down | 5 (16) | 55.7 | 5.74 | 0.01 | 0.01 | 0.01 |
| A0A061FFU2 | Xylem serine proteinase 1, putative isoform 1 | Serine-type endopeptidase activity | ns | ns | ↑Up | 9 (20) | 80.6 | 6.33 | 0.42 | 0.94 | 100.00 |
| A0A061DXF3 | 2-oxoglutarate and Fe(II)-dependent oxygenase superfamily protein | Protein metabolic process/ acid nucleic repair and/or modification | ↑Up | ns | ns | 1 (5) | 46.5 | 6.54 | 100.00 | 0.72 | 0.75 |
| A0A061GTX8 | Alpha/beta-Hydrolases superfamily protein | Hydrolase activity | ↑Up | ns | ns | 20 (73) | 36.6 | 6.14 | 5.47 | 1.69 | 2.31 |
| A0A061E2W9 | Alpha/beta-Hydrolases superfamily protein isoform 2 | Hydrolase activity | ↓Down | ↓Down | ↓Down | 5 (18) | 35.1 | 5.63 | 0.16 | 0.15 | 0.09 |
| A0A061DI19 | Asparagine synthetase [glutamine-hydrolyzing] | Asparagine synthase activity | ↑Up | ns | ns | 2 (6) | 65.9 | 6.68 | 100.00 | 0.57 | 0.37 |
| A0A061FQC7 | Map3k delta-1 protein kinase, putative isoform 1 | Protein Serine/threonine kinase | ↑Up | ns | ns | 1 (1) | 84.4 | 6.79 | 3.29 | 1.27 | 1.25 |
| A0A061H034 | Ubiquitin-conjugating enzyme 13 isoform 1 | Protein polyubiquitination | ↓Down | ↓Down | ↓Down | 1 (9) | 26.4 | 5.36 | 0.01 | 0.01 | 0.01 |
| A0A061EYD4 | Ubiquitinyl hydrolase 1 | Protein deubiquitination | ↓Down | ↓Down | ↓Down | 4 (5) | 131.2 | 6.01 | 0.01 | 0.01 | 0.01 |
| A0A061ENB9 | Acylaminoacyl-peptidase | Serine-type endopeptidase activity | ↓Down | ns | ns | 4 (8) | 85.6 | 6.49 | 0.32 | 0.40 | 0.40 |
| A0A061G4K9 | Peptidase M1 family protein | Metallopeptidase activity | ↓Down | ↓Down | ↓Down | 3 (6) | 116.4 | 6.89 | 0.01 | 0.01 | 0.01 |
| A0A061GAK7 | Eukaryotic aspartyl protease family protein | Aspartic-type endopeptidase activity | ↓Down | ↓Down | ↓Down | 2 (5) | 52.4 | 8.6 | 0.01 | 0.01 | 0.01 |
| A0A061E6C8 | Glutathione synthetase | Glutathione synthase activity | ↓Down | ↓Down | ↓Down | 3 (8) | 63.2 | 8.35 | 0.01 | 0.01 | 0.01 |
| A0A061EL40 | NEDD8-activating enzyme E1 catalytic subunit | NEDD8 activating enzyme activity | ↓Down | ↓Down | ↓Down | 2 (8) | 49.4 | 5.67 | 0.01 | 0.01 | 0.01 |
| A0A061DI13 | Serine hydroxymethyltransferase | Glycine hydroxymethyltransferase activity | - | ↓Down | ↓Down | 9 (31) | 47.1 | 8.95 | - | 0.01 | 0.01 |
| A0A061DKU6 | Serine/threonine protein phosphatase 2A 55 kDa regulatory subunit B prime gamma, putative isoform 2 (Fragment) | Protein binding | ↓Down | ↓Down | ↓Down | 1 (8) | 33.7 | 5.55 | 0.01 | 0.01 | 0.01 |
| A0A061E5N4 | Serine/threonine-protein phosphatase | Catalytic activity | ↓Down | ↓Down | ↓Down | 3 (18) | 35 | 4.96 | 0.01 | 0.01 | 0.01 |
| A0A061DI67 | FAD-dependent oxidoreductase family protein | Oxidoreductase activity | ↓Down | ↓Down | ↓Down | 2 (6) | 45.1 | 6.92 | 0.01 | 0.01 | 0.01 |
| A0A061GWD1 | SELT-like protein | Cell redox homeostasis | - | - | ↓Down | 1 (8) | 25 | 8.29 |  |  | 0.01 |
| A0A061GC11 | Pyridoxal phosphate (PLP)-dependent transferases superfamily protein isoform 1 | Methionine-oxo-acid transaminase activity | ↓Down | ↓Down | - | 2 (9) | 43.7 | 6.55 | 0.01 | 0.01 |  |
| A0A061F345 | Peptide n-glycanase, putative isoform 1 | Glycopeptidase activity | - | ↓Down | ↓Down | 2 (6) | 82.6 | 5.86 |  | 0.01 | 0.01 |
| A0A061GFH4 | Methionine S-methyltransferase | Methionine S-methyltransferase activity | ↓Down | ↓Down | ↓Down | 1 (1) | 121 | 5.82 | 0.01 | 0.01 | 0.01 |
|  |  |  |  |  |  |  |  |  |  |  |  |
| **Carbohydrate metabolic process** | |  |  |  |  |  |  |  |  |  |  |
| A0A061G1D5 | Chitinase | Glycosidase activity | ↑Up | ↑Up | ns | 8 (32) | 32 | 9.69 | 6.19 | 4.12 | 4.20 |
| A0A061DUS2 | Chitinase | Glycosidase activity | ↑Up | ns | ns | 7 (26) | 31.5 | 4.54 | 5.65 | 2.63 | 2.30 |
| A0A061FYK7 | Chitinase | Glycosidase activity | ns | ↑Up | ↑Up | 2 (7) | 32.7 | 4.32 | 1.78 | 100.00 | 100.00 |
| A0A061EPY8 | Glucan endo-1,3-beta-D-glucosidase | Hydrolase activity | ↑Up | ↑Up | ↑Up | 5 (18) | 46.6 | 6.95 | 9.58 | 100.00 | 100.00 |
| A0A061F3N5 | Glucan endo-1,3-beta-D-glucosidase | Hydrolase activity | ↑Up | ns | ns | 26 (69) | 40.9 | 9.19 | 2.86 | 0.71 | 0.54 |
| A0A061DF66 | Basic chitinase | Chitinase activity | ns | ↑Up | ns | 5 (34) | 33.5 | 7.97 | 1.10 | 5.51 | 3.24 |
| A0A061DXH0 | Basic chitinase | Chitinase activity | ns | ns | ↑Up | 13 (67) | 34.8 | 5.48 | 2.15 | 1.68 | 4.72 |
| A0A061DGD0 | Basic chitinase | Chitinase activity | ↓Down | ns | ns | 17 (68) | 28.8 | 6.33 | 9.13 | 0.59 | 0.85 |
| A0A061GET9 | Chitinase family protein | Chitinase activity | ns | ↑Up | ↑Up | 6 (36) | 35.6 | 6.9 | 1.31 | 5.76 | 7.35 |
| A0A061G0U1 | Sugar isomerase (SIS) family protein isoform 2 | Isomerase activity | ↑Up | ns | ns | 7 (73) | 17.7 | 7.03 | 3.09 | 1.80 | 2.07 |
| A0A061FWV6 | Ketose-bisphosphate aldolase class-II family protein isoform 1 | Oxidoreductase activity | ↑Up | ↓Down | ↓Down | 2 (2) | 147.4 | 6.46 | 100.00 | 0.17 | 0.20 |
| A0A061DKM8 | Xyloglucan endotransglucosylase/hydrolase | Endoxyloglucan transferase activity | ↑Up | ns | ns | 3 (12) | 33.5 | 8 | 100.00 | 0.35 | 0.48 |
| A0A061EK18 | Carrot EP3-3 chitinase, putative isoform 1 | Chitinase activity | ↑Up | ns | ns | 13 (51) | 36.7 | 8.76 | 2.75 | 2.36 | 3.86 |
| A0A061GD78 | Beta-galactosidase | Beta-galactosidase activity | ns | - | ↑Up | 4 (9) | 93.5 | 7.77 | 0.89 |  | 100.00 |
| A0A061FUY0 | Amidase family protein isoform 1 | Amidase activity | ns | ns | ↑Up | 2 (5) | 64.9 | 8.18 | 1.13 | 1.11 | 100.00 |
| A0A061G3R6 | Starch synthase, chloroplastic/amyloplastic | Glycogen (starch) synthase activity | ns | ↑Up | ↑Up | 3 (10) | 67.1 | 8.4 | 1.24 | 100.00 | 100.00 |
| A0A061ENU3 | Periplasmic beta-glucosidase, putative | Xylan 1,4-beta-xylosidase activity | ns | ns | ↑Up | 4 (7) | 83.9 | 8.6 | 1.28 | 1.91 | 100.00 |
| A0A061EK23 | Carrot EP3-3 chitinase | Chitinase activity | ns | ns | ↑Up | 5 (27) | 28.8 | 5.14 | 2.67 | 1.00 | 9.01 |
| A0A061FZ71 | 1,3-beta-glucan synthase | Glucosyltransferase activity | ↓Down | ↓Down | ↓Down | 2 (2) | 218.1 | 8.12 | 0.01 | 0.01 | 0.01 |
| A0A061GHV7 | Arabinose kinase isoform 1 | L-arobinokinase activity | ↓Down | ↓Down | ↓Down | 4 (6) | 108.9 | 6.46 | 0.01 | 0.01 | 0.01 |
| A0A061E9Z0 | Alpha-1,4 glucan phosphorylase | Glycosyltransferase | ↓Down | ↓Down | - | 4 (6) | 105.7 | 6 | 0.01 | 0.01 |  |
| A0A061E5Y7 | Glucose-6-phosphate 1-dehydrogenase | Glucose-6-phosphate dehydrogenase activity | ↓Down | ↓Down | ↓Down | 2 (4) | 65.6 | 7.33 | 0.01 | 0.01 | 0.01 |
| A0A061ECX9 | Phosphoglycerate mutase (2,3-diphosphoglycerate-independent) | Phosphoglycerate mutase activity | ↓Down | ↓Down | ↓Down | 5 (16) | 61.7 | 628 | 0.01 | 0.01 | 0.01 |
| A0A061GD94 | Glycosyl hydrolases family 32 protein isoform 1 | O-glucosyl hydrolase activity | ↓Down | ↓Down | ↓Down | 9 (26) | 64.7 | 8.29 | 0.01 | 0.01 | 0.01 |
| A0A061DMV2 | Polygalacturonase | Polygalacturonase activity | ↓Down | ns | ns | 9 (29) | 43.3 | 8.62 | 0.22 | 0.75 | 0.71 |
|  |  |  |  |  |  |  |  |  |  |  |  |
| **Secondary metabolites biosynthesis** | |  |  |  |  |  |  |  |  |  |  |
| ***Terpenoid Biosynthesis*** | |  |  |  |  |  |  |  |  |  |  |
| A0A061GF49 | 3S-linalool/(E)-nerolidol /(E,E)-geranyl linalool synthase | Terpene synthase activity | ↓Down | ↓Down | ns | 8 (39) | 21.8 | 6.28 | 0.27 | 0.01 | 1.03 |
| A0A061GGG2 | (+)-delta-cadinene synthase | (+)-delta-cadinene synthase activity | ↓Down | ↓Down | ns | 21 (39) | 67 | 6.15 | 0.34 | 0.03 | 0.70 |
| A0A061FR70 | Terpene cyclase/mutase family member | Isomerase activity | ↓Down | ↓Down | ↓Down | 7 (13) | 85.6 | 6.15 | 0.01 | 0.01 | 0.01 |
| A0A061ENM7 | Cytochrome P450, family 81, subfamily D, polypeptide 8, putative | Heme binding | ns | ↓Down | ↓Down | 1 (3) | 61.7 | 8.59 | 0.44 | 0.16 | 0.21 |
| A0A061GYL3 | 4-hydroxy-3-methylbut-2-enyl diphosphate synthase isoform 1 | Terpene synthase activity | ↓Down | ↓Down | ↓Down | 4 (8) | 82.1 | 6.51 | 0.01 | 0.01 | 0.01 |
|  | |  |  |  |  |  |  |  |  |  |  |
| ***L-phenylalanine degradation*** | |  |  |  |  |  |  |  |  |  |  |
| A0A061DJ69 | Cinnamyl alcohol dehydrogenase 9 isoform | Cinnamyl-alcohol dehydrogenase activity | ↑Up | ns | ↑Up | 20 (77) | 38.9 | 7.23 | 4.01 | 4.31 | 8.70 |
| A0A061DIQ8 | Alcohol dehydrogenase 1 | Alcohol dehydrogenase activity | ns | ns | ↓Down | 9 (28) | 41.3 | 6.6 | 0.43 | 0.53 | 0.11 |
| A0A061G9C7 | Cinnamoyl coa reductase 1 isoform 1 | Oxidoreductase activity | ns | ↓Down | ns | 11 (49) | 36.9 | 6.46 | 0.90 | 0.17 | 0.85 |
| A0A061F0U3 | Benzoyl coenzyme A: Benzyl alcohol benzoyl transferase | Transferases | ↓Down | ↓Down | ↓Down | 6 (22) | 50.5 | 7.01 | 0.01 | 0.01 | 0.01 |
| A0A061E210 | Short chain alcohol dehydrogenase | NAD(P)(H) dependent oxidoreductase | ↓Down | ↓Down | ns | 13 (77) | 28.4 | 6.52 | 0.17 | 0.09 | 0.87 |
| A0A061E215 | Short chain alcohol dehydrogenase | NAD(P)(H) dependent oxidoreductase | ↓Down | ↓Down | ↓Down | 9 (52) | 28.4 | 6.86 | 0.01 | 0.01 | 0.01 |
|  |  |  |  |  |  |  |  |  |  |  |  |
| ***Proanthocyanin biosynthesis*** | |  |  |  |  |  |  |  |  |  |  |
| A0A061FTJ1 | Chalcone-flavonone isomerase family protein | Isomerase activity | ns | ↑Up | ns | 3 (19) | 29.4 | 5.02 | 1.21 | 100.00 | 1.33 |
| A0A061E9U7 | UDP-glucosyl transferase 88A1, putative | Transferase activity | ↑Up | ns | ↑Up | 3 (7) | 50.8 | 6.37 | 100.00 | 1.28 | 100.00 |
| A0A061G577 | Leucoanthocyanidin dioxygenase isoform 1 | 2-oxoglutarate-dependent dioxygenase activity | ↓Down | - | ↓Down | 2 (12) | 43.3 | 6.3 | 0.01 |  | 0.01 |
| ***Others Secondary Metabolism*** | |  |  |  |  |  |  |  |  |  |  |
| S1RU27 | Secretory laccase isoform 2 | Oxidoreductase activity | ↑Up | ↑Up | ↑Up | 2 (9) | 39 | 6.33 | 4.40 | 100.00 | 6.13 |
| S1SMV4 | Secretory laccase, putative isoform 1 | Oxidoreductase activity | ↑Up | ns | ns | 1 (4) | 32.4 | 5.9 | 5.88 | 3.00 | 3.50 |
| A0A061FKJ6 | Laccase | Oxidoreductase activity | ↑Up | ↑Up | ↑Up | 4 (12) | 62.3 | 5.16 | 14.75 | 9.39 | 8.33 |
| A0A061GTT4 | Strictosidine synthase-like 3 | Strictosidine synthetase activity | ↓Down | ns | ns | 13 (45) | 43.7 | 6.73 | 0.31 | 0.59 | 0.42 |
|  |  |  |  |  |  |  |  |  |  |  |  |
| **Lipid biosynthetic and metabolic process** | |  |  |  |  |  |  |  |  |  |  |
| A0A061EKE7 | AMP-dependent synthetase and ligase family protein isoform 3 | Ligase activity | ↓Down | ↓Down | ↓Down | 4 (14) | 59.6 | 7.66 | 0.01 | 0.01 | 0.01 |
| A0A061EY43 | 1-acylglycerol-3-phosphate O-acyltransferase | Transferase activity | ↓Down | ↓Down | ↓Down | 2 (8) | 45.1 | 9.38 | 0.01 | 0.01 | 0.01 |
| A0A061EPV8 | Acyl carrier protein | Acyl carrier activity | ns | ns | ↑Up | 1 (11) | 14.6 | 4.94 | 0.95 | 1.48 | 100.00 |
| A0A061FG41 | GDSL-like Lipase/Acylhydrolase superfamily protein | Hydrolase activity | ↓Down | ns | ns | 7 (30) | 43.5 | 7.42 | 0.22 | 0.60 | 0.60 |
| A0A061F5Q5 | GDSL-like Lipase/Acylhydrolase superfamily protein, putative | Hydrolase activity | ns | ↑Up | ↑Up | 2 (5) | 87.8 | 5.49 | 0.69 | 100.00 | 100.00 |
| A0A061GQZ4 | GDSL-like Lipase/Acylhydrolase superfamily protein, putative | Hydrolase activity | ↑Up | ns | ns | 6 (23) | 41.9 | 4.49 | 3.20 | 1.00 | 0.96 |
| A0A061EC84 | GDSL-motif lipase/hydrolase 6 | Hydrolase activity | ns | ns | ↑Up | 1 (3) | 39.9 | 8.31 | 1.65 | 4.44 | 100.00 |
| A0A061DSG1 | Long-chain-fatty-acid--CoA ligase | Long-chain fatty acid-CoA ligase activity | ↓Down | ↓Down | ↓Down | 2 (5) | 74.2 | 6.96 | 0.01 | 0.01 | 0.01 |
| A0A061F3Q9 | Lipoxygenase | Oxireductase activity | ns | ns | ↓Down | 18 (18) | 98.3 | 7.27 | 1.37 | 0.13 | 0.59 |
| A0A061EKX2 | Cytochrome B5, n4,ATCB5-B,CB5-B | Heme binding | - | ↓Down | ↓Down | 2 (26) | 14.7 | 5.53 |  | 0.01 | 0.01 |
| A0A061GL75 | Sulfoquinovosyldiacylglycerol 1 | UDPsulfoquinovose synthase activity | ↑Up | ns | ns | 1 (3) | 53.7 | 8.21 | 3.02 | 1.99 | 2.84 |
| A0A061DR75 | ATP citrate synthase | Lyase activity | ↓Down | ↓Down | ↓Down | 13 (31) | 65.8 | 7.69 | 0.01 | 0.01 | 0.01 |
| A0A061F5J0 | Zinc finger protein, putative | Esterase activity | ns | ns | ↑Up | 2 (7) | 40.3 | 8.57 | 1.06 | 1.23 | 100.00 |
|  |  |  |  |  |  |  |  |  |  |  |  |
| **Defense and Stress mechanisms** | |  |  |  |  |  |  |  |  |  |  |
| A0A061GXF3 | HSP20-like chaperones superfamily protein | Protein binding | ↓Down | ↓Down | - | 10 (67) | 17.3 | 6.6 | 0.01 | 0.01 |  |
| A0A061GXF6 | HSP20-like chaperones superfamily protein | Protein binding | ↓Down | ↓Down | ↓Down | 6 (32) | 18.1 | 6.37 | 0.01 | 0.01 | 0.01 |
| A0A061EQN0 | HSP20-like chaperones superfamily protein, putative | Protein binding | ns | ns | ↑Up | 1 (6) | 17.4 | 8.12 | 1.70 | 1.24 | 100.00 |
| A0A061DWT3 | Basic pathogenesis-related protein 1 | Pathogen defense | ns | ns | ↑Up | 1 (5) | 24.2 | 5.24 | 2.53 | 0.67 | 8.77 |
| A0A061G549 | Osmotin 34 | Pathogen defense | ↑Up | ns | ns | 7 (40) | 26.1 | 5.87 | 4.23 | 4.42 | 3.13 |
| A0A061G5M4 | Osmotin 34 | Pathogen defense | ↑Up | ↑Up | ns | 10 (55) | 24.4 | 8.06 | 50.70 | 48.63 | 2.11 |
| A0A061GVG5 | Early nodulin-like protein 14, putative | Electron transfer activity | ↑Up | ns | ↑Up | 1 (5) | 17.4 | 5.63 | 5.61 | 2.91 | 100.00 |
| A0A061GJC7 | Early nodulin-like protein 15, putative | Electron transfer activity | ns | ↑Up | ns | 5 (29) | 19.5 | 7.85 | 1.18 | 7.03 | 3.86 |
| A0A061E492 | Early nodulin-like protein 3, putative | Electron transfer activity | ns | ↓Down | ↓Down | 5 (18) | 27.7 | 9.42 | 2.00 | 4.52 | 6.33 |
| A0A061E3B7 | Peroxidase | Heme binding | ns | ns | ↑Up | 3 (12) | 35.5 | 8.79 | 2.62 | 2.89 | 100.00 |
| A0A061FZ04 | Peroxidase | Heme binding | ns | ns | ↑Up | 1 (6) | 34.7 | 9.22 | 0.97 | 1.07 | 100.00 |
| A0A061DNW7 | Peroxidase | Heme binding | ↑Up | ns | ns | 12 (57) | 35.5 | 8.7 | 3.76 | 2.44 | 3.25 |
| A0A061E2H9 | Peroxidase | Heme binding | ↑Up | ↑Up | ↑Up | 12 (33) | 54.5 | 9.26 | 9.17 | 7.54 | 5.95 |
| A0A061FHR8 | Peroxidase | Heme binding | ns | ns | ↑Up | 5 (28) | 31.1 | 8.37 | 0.61 | 1.14 | 100.00 |
| A0A061GSL9 | Peroxidase | Heme binding | ns | ns | ↑Up | 6 (25) | 37.1 | 4.78 | 1.30 | 3.52 | 4.33 |
| A0A061E9G4 | MLP-like protein 28 | Catalytic activity | ↑Up | ↑Up | ↑Up | 2 (17) | 17.6 | 5.53 | 100.00 | 100.00 | 100.00 |
| A0A061F0C6 | MLP-like protein 423, putative | Catalytic activity | ↑Up | ↑Up | ↑Up | 3 (32) | 17.3 | 4.73 | 5.59 | 5.50 | 7.63 |
| A0A061GL74 | Senescence-related gene 1 | Oxidoreductase activity | ns | ns | ↑Up | 3 (13) | 41.6 | 5.83 | 1.17 | 0.71 | 100.00 |
| A0A061E155 | Plant basic secretory protein (BSP) family protein | Unknown | ↑Up | ns | ↑Up | 2 (9) | 25.5 | 6.89 | 4.14 | 1.56 | 10.53 |
| A0A061E9D1 | Plant EC metallothionein-like protein, putative | Zinc ion binding | ↑Up | ↑Up | ↑Up | 1 (18) | 7.2 | 6.95 | 100.00 | 41.19 | 100.00 |
| A0A061G576 | Glutathione peroxidase | Glutatione peroxidase activity | ↓Down | ↓Down | ↓Down | 1 (8) | 19.4 | 5.39 | 0.01 | 0.01 | 0.01 |
| A0A061G8E4 | Heat shock protein 90.1 isoform 1 | Binding | - | ↓Down | ↓Down | 27 (36) | 80.5 | 5.1 |  | 0.01 | 0.01 |
| A0A061EJV5 | Phosphoenolpyruvate carboxylase family protein isoform 5 (Fragment) | Catalytic activity | ↓Down | ↓Down | ↓Down | 1 (2) | 49.2 | 8.66 | 0.01 | 0.01 | 0.01 |
| A0A061F1P4 | Late embryogenesis abundant (LEA) hydroxyproline-rich glycoprotein family, putative | Antimicrobial humoral response | ns | ↑Up | ↑Up | 3 (23) | 25.4 | 9.25 | 2.16 | 100.00 | 100.00 |
| A0A061EXA1 | Polygalacturonase inhibitor | Cell wall proteins | ns | ↑Up | Ns | 4 (18) | 37 | 7.36 | 2.08 | 100.00 | 1.85 |
| A0A061GXK4 | Senescence-associated gene 13 isoform 1 | Oxidoreductase activity | ↑Up | ns | Ns | 9 (30) | 30.6 | 6.54 | 2.93 | 2.44 | 2.38 |
| A0A061G691 | Elicitor-responsive protein, putative | Unknown | ↓Down | ns | Ns | 2 (16) | 16.6 | 4.44 | 0.20 | 0.19 | 0.43 |
| A0A061DNF1 | Plasmodesmata callose-binding protein 5 | Unknown | ↓Down | - | - | 1 (9) | 18.3 | 5.4 | 0.01 |  |  |
| A0A061EJM1 | Scorpion toxin-like knottin superfamily protein | Killing by host of symbiont cells | ↑Up | ns | ↓Down | 2 (11) | 15.9 | 8.81 | 100.00 | 1.08 | 0.15 |

* Up-regulated proteins were defined by an abundance ratio ≥ 2.0, and down-regulated proteins by a ratio ≤ 0.5, with an adjusted p-value cutoff of ≤ 0.001. Proteins present exclusively in one genotype (on/off) had their ratios arbitrarily set to 100 or 0.01.
